# Identification of Key Gene Networks and Deciphering Transcriptional Regulators Associated With Peanut Embryo Abortion Mediated by Calcium Deficiency

**DOI:** 10.3389/fpls.2022.814015

**Published:** 2022-03-21

**Authors:** Hua Chen, Qiang Yang, Huiwen Fu, Kun Chen, Shanshan Zhao, Chong Zhang, Tiecheng Cai, Lihui Wang, Wenzhi Lu, Hao Dang, Meijia Gao, Huaqi Li, Xinyi Yuan, Rajeev K. Varshney, Weijian Zhuang

**Affiliations:** ^1^Key Laboratory of Ministry of Education for Genetics, Breeding and Multiple Utilization of Crops, Institute of Oil Crops Research, Research Center for Genetics and Systems Biology of Leguminous Oil Plants, Fujian Agriculture and Forestry University, Fuzhou, China; ^2^State Key Laboratory of Ecological Pest Control for Fujian and Taiwan Crops, Fujian Agriculture and Forestry University, Fuzhou, China; ^3^State Agricultural Biotechnology Center, Center for Crop and Food Innovation, Food Futures Institute, Murdoch University, Murdoch, WA, Australia

**Keywords:** embryo abortion, calcium, hormone, WGCNA, *Arachis hypogaea* L.

## Abstract

Peanut embryo development is easily affected by a variety of nutrient elements in the soil, especially the calcium level. Peanut produces abortive embryos in calcium-deficient soil, but underlying mechanism remains unclear. Thus, identifying key transcriptional regulators and their associated regulatory networks promises to contribute to a better understanding of this process. In this study, cellular biology and gene expression analyses were performed to investigate peanut embryo development with the aim to discern the global architecture of gene regulatory networks underlying peanut embryo abortion under calcium deficiency conditions. The endomembrane systems tended to disintegrate, impairing cell growth and starch, protein and lipid body accumulation, resulting in aborted seeds. RNA-seq analysis showed that the gene expression profile in peanut embryos was significantly changed under calcium deficiency. Further analysis indicated that multiple signal pathways were involved in the peanut embryo abortion. Differential expressed genes (DEGs) related to cytoplasmic free Ca^2+^ were significantly altered. DEGs in plant hormone signaling pathways tended to be associated with increased IAA and ethylene but with decreased ABA, gibberellin, cytokinin, and brassinosteroid levels. Certain vital genes, including apoptosis-inducing factor, WRKYs and ethylene-responsive transcription factors, were up-regulated, while key regulators of embryo development, such as *TCP4*, *WRI1*, *FUS3*, *ABI3*, and *GLK1* were down-regulated. Weighted gene co-expression network analysis (WGCNA) identified 16 significant modules associated with the plant hormone signaling, MAPK signaling, ubiquitin mediated proteolysis, reserve substance biosynthesis and metabolism pathways to decipher regulatory network. The most significant module was darkolivegreen2 and *FUS3* (AH06G23930) had the highest connectivity among this module. Importantly, key transcription factors involved in embryogenesis or ovule development including *TCP4*, *GLK1*, *ABI3*, *bHLH115*, *MYC2*, etc., were also present in this module and down regulated under calcium deficiency. This study presents the first global view of the gene regulatory network involved in peanut embryo abortion under calcium deficiency conditions and lays foundation for improving peanut tolerances to calcium deficiency by a targeted manipulation of molecular breeding.

## Introduction

Peanut (*Arachis hypogaea* L.) is a major agronomic crop providing important sources of plant oil and proteins worldwide. Peanut productivity has been adversely challenged by various biotic and abiotic stresses, leading to a significant reduction (Pandey et al., 2012).

Peanut embryo development is easily affected by a variety of nutrient elements in the soil, especially the calcium level. Calcium is a universal second messenger and essential mineral nutrient for plant growth and development. Peanut is a calcium-addicted crop. Calcium deficiency can seriously affect peanut growth and development and causes a series of physiological changes in peanut. Young leaves turn yellow in older tissues owing to the immobility of Ca^2+^. The number of flowers increases during anthesis, but most flowers are aborted. The mature leaves in the bottom outer layer form atrophic lesions in the late growth stage, vacuolar membrane of mesophyll cells breaks, chloroplast expands loosely, capsule breaks, and grana lamella structure is destroyed, which accelerates leaf senescence (Lin, 1996). Necrosis occurred at bud tip and root tip under the extreme calcium deficiency condition. The cell wall is loose and distorted and plasmolysis occurred. More than 90% calcium is directly absorbed from the soil by the peanut pod during development (Beringer and Taha, 1976). Peanuts growing in calcium-deficient soil produce a high rate of abortive embryos with empty or defectively filled pods, resulting in great economic losses (Jain et al., 2011) which poses a serious problem for global peanut production, especially in tropical and sub-tropical areas. Fortunately, application of calcium fertilizer could significantly increase the pod yield of peanut in acid soil (Zhang et al., 2015). Zhang has reported that peanut yields can increase by 26.92% with 210 kg ha^–1^ fused CaO treatment, which might be related to higher pod numbers per plant, higher double kernel rate, and higher plumpness of kernel under CaO treatment (Zhang et al., 2015). Thilakarathna also reported that the addition of 250 kg ha^–1^ of gypsum can increase the pod dry weight of peanuts by 39% (Thilakarathna et al., 2015). In addition to poor peanut yield and quality (Murata et al., 2008), calcium deficiency deteriorates seed viability and germination.

Different cultivars showed diverse sensitivities to calcium (Wu H. et al., 2017). Large pod varieties tend to produce a much higher proportion of empty pods in calcium-deficient soil. Tropical and subtropical areas thus use only peanut cultivars of small pod with yield performance reductions by approximately half compared with the large pod varieties. In China, more than 1.1 million hectares were planted with peanut in the south of peanut production regions. However, the yield per unit area of peanut is much lower than the national average, which is mainly due to the lack of calcium in the soil. It is of great significance to elucidate the mechanism controlling peanut embryo abortion for molecular breeding of large pod varieties with higher tolerance to low calcium. To address this important biological problem, different methods such as DDRT-PCR (Zhang et al., 2008), SSHaLL (Chen et al., 2016), proteomics (Zhang et al., 2007) and microRNA analysis (Chen et al., 2019) have been employed and various essential genes identified (Jain et al., 2011), providing initial insights into the mechanism of low calcium-induced embryo abortion.

With the rapid development of next-generation sequencing technologies, transcriptome profiling is a powerful approach for generating a transcriptional map at the whole-genome scale to discern regulatory networks and has been widely applied to plants such as peanut for screening candidate genes involved in plant growth and development as well as response to abiotic and biotic stresses (Xia et al., 2013). Weighted gene correlation network analysis (WGCNA) is an effective and robust network modeling method, which can identify co-expression modules regulating plant development and responsive to various stresses (Zhang and Horvath, 2005).

In this study, we firstly investigated the cell structure and morphology changes of peanut embryos under calcium deficiency conditions at the stage of 15, 20, and 30 DAP (days after pegging) using transmission electronic microscopy (TEM). Then global gene expression atlas showing genome-wide expression of genes in peanut embryos under calcium deficiency were developed from RNA-seq data to identify the key regulated genes in embryo abortion. Furthermore, WGCNA analysis was employed to comprehensively identify important regulators or pathways contributing to peanut abortion under calcium deficiency conditions. The results will further our understanding of dissecting the networks regulating calcium deficiency-induced embryo abortion and provide insights into mechanisms responsible for embryo development, especially its abortion under calcium deficiency. It will also lay the foundation for peanut molecular breeding of large pod varieties with higher tolerance to low calcium.

## Materials and Methods

### Plant Materials and Calcium Treatment

Plant material was collected from peanut cultivar MH6 grown in Ca^2+^-deficient soil in Pingtan, Fujian Province of China. The exchangeable Ca^2+^ content in the soil was 0.6 cmol/kg soil. Peanuts grown in this soil were used as the Ca^2+^ deficiency-treated material, and peanuts grown in the same soil fertilized with 75 kg/667 m^2^ plaster (CaO) were used as the Ca^2+^ sufficiency-treated material. The exchangeable Ca^2+^ content after fertilization was 4.2 cmol/kg soil, and generally the critical value of Ca^2+^ content in soil that could result in peanut embryo abortion was < 3.0 cmol/kg soil (Zhang, 2004). Embryos (15, 20, and 30 DAP) were manually dissected, frozen in liquid nitrogen and stored at −80°C for RNA-seq and qRT-PCR. Three biological replicates were prepared for all treatments.

### Transmission Electronic Microscopy Observation for Peanut Embryo Cells

Peanut embryos (15, 20, and 30 DAP) were fixed in 5% (v/v) glutaric dialdehyde in 0.1 M phosphate buffer (pH 7.3) for 24 h at 4°C and postfixed in 1% (w/v) osmic acid for 1–2 h. The embryos were dehydrated using a graded ethanol series and embedded in ERL-4206. The embedded embryos were then placed in an incubator chamber at 70°C for polymerization for 8–10 h. The specimens were sectioned to a thickness of 50–70 nm, and the sections were moved to a copper screen coated with a thin film. The sections were stained with both uranyl acetate and lead citrate to observe the ultrastructure of peanut embryos using TEM.

### RNA Sequencing and Data Analysis

Total RNA was extracted from calcium deficiency-treated and control peanut embryos with TRIzol reagent (Invitrogen, Carlsbad, CA, United States). The RNAs were treated with RNase-free DNase I (Takara, Dalian, China) to eliminate contaminating genomic DNA. cDNA libraries were prepared using Illumina Paired End Sample Prep Kit. Three biological replicates were performed for each library. The quality of the libraries was evaluated using a Qubit2.0 Fluorometer (Life Technologies, CA, United States) and Agilent Bioanalyzer 2100 system (Agilent Technologies, CA, United States). RNA-seq libraries were sequenced *via* Illumina HiSeq™ 2500 platform (BIOMARKER, China). Raw reads were filtered to discard adapter sequences, low-quality reads (including reads containing more than > 10% poly-N and reads with more than 50% bases with a *Q* value ≤ 10). Filtered reads were mapped to the cultivated peanut reference genome^1^ (Zhuang et al., 2019) using the Bowtie tools software v.1.0.0 (Langmead et al., 2009) and TopHat2 (Kim et al., 2013) for reporting all mapping locations. The mapped reads were assembled by Cufflinks (Trapnell et al., 2010). Fragments per Kilobase of exon per Million Fragments (FPKM) was used to measure Cuffdiff to describe the transcript sufficiency. DESeq software (version 1.18.0) was applied with a criterion of a | log_2_fold change| ≥ 1 and FDR (false discovery rate) < 0.01 (Anders and Huber, 2010) to detect differentially expressed genes (DEGs).

The differentially expressed genes were subjected to NR, Swiss-Prot, GO, and COG to predict their biological functions. In addition, DEGs were subjected to the KEGG, KOG and Pfam databases to perceive their biological roles. KOBAS software (version 2.0) was used to test the statistical enrichment of differentially expressed genes in KEGG pathways. GO enrichment analysis was implemented with a *p* ≤ 0.05 as the threshold, and topGO was used to generate the acyclic graph.

### Regulatory Network Construction by Weighted Gene Co-correlation Network Analysis

Weighted gene co-correlation network analysis (WGCNA) was performed to construct gene co-expression networks using WGCNA R (version 4.0.3) package (Langfelder and Horvath, 2008). Genes with low abundance (FPKM value < 0.5) were filtered to eliminate noise. All genes were used as input to the signed WGCNA network construction. The co-expression modules were built using the automatic network construction function block wise Modules with default settings, except that the soft power was set to 30, merge Cut Height value was 0.25 and min Module Size was 30. The network of modules of co-expressed genes (edge weight > 0.15) was visualized by Cytoscape 3.9.0 (Saito et al., 2012).

### Quantitative Real-Time PCR Validation for Differentially Expressed Genes

Real-time PCR for the relative expression level of DEGs was performed using ChamQ SYBR qPCR Master Mix (High ROX Premixed) (Vazyme, Nanjing, China) with specific primers (Supplementary Table 11), and *Ahactin* was used as an internal reference gene. All reactions were performed on an ABI7500 system in triplicate. The relative expression levels of the DEGs were calculated using the comparative CT method (2^–△^^△^ *^CT^* method) (Schmittgen and Livak, 2008), followed by normalization to the PCR threshold cycle number (Ct value) of the DEGs to that of the reference gene. The Student’s *t*-test was employed to compare differences between the control and experimental values.

## Results

### Ca^2+^ Deficiency Deteriorated Peanut Embryo Cell and the Reserve Accumulation

**FIGURE 1:**
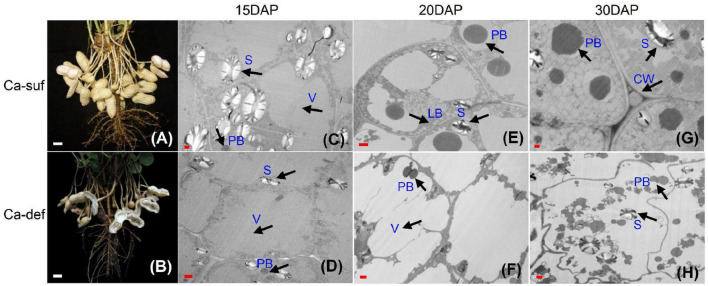
Effects of calcium on peanut pod development and embryo cell characteristics. **(A)** Peanut pods grown under calcium sufficiency conditions at 30 DAP. **(B)** Peanut pods grown under calcium deficiency conditions at 30 DAP. **(C)** Embryo cell of 15 DAP with calcium sufficiency. **(D)** Embryo cell of 15 DAP with calcium deficiency. **(E)** Embryo cell of 20 DAP with calcium sufficiency. **(F)** Embryo cell of 20 DAP with calcium deficiency. **(G)** Embryo cell of 30 DAP with calcium sufficiency; **(H)** Embryo cell of 30 DAP with calcium deficiency. LB, lipid body; PB, protein body; N, nucleus; S, starch; V, vacuole; CW, cell wall. Bars indicated in panels **(A,B)** are 10 mm; Bars in panels **(C,D)** are 1 μm and Bars in panels **(E,F)** are 0.1 μm.

Our previous study indicated that peanut pods at 15, 20 and 30 days after pegging (DAP) tended to be aborted and reduced the peanut yield and quality (Chen et al., 2019). Here, the embryo cells were investigated using transmission electronic microscopy (TEM) at 15, 20 and 30 DAP under calcium deficiency and sufficiency conditions. No obvious differences in cell morphology at 15 DAP were observed, except that the calcium-deficient cells contained fewer and smaller starch granules in the cytoplasm. The embryo cells contained large central vacuoles and thin cell walls at this stage (Figures 1C,D). At 20 DAP, the cell wall was clearly thickening under calcium sufficiency condition, while the calcium deficiency embryo cells remained thinner (Figures 1E,F). Moreover, the central vacuoles were compartmented into smaller ones surrounded by cytoplasm under calcium sufficiency. More vacuole proteins with different sizes were developed inside the vacuoles. Small lipid bodies began to form between the vacuoles and inside the cell walls (Figure 1E). Nevertheless, no clear developmental events occurred in calcium deficiency embryo cells. Their endomembrane systems appeared somewhat disintegrated, with a large reduction of lipid bodies inside the cells (Figure 1F). At 30 DAP, the embryo cell morphology with deficient calcium stress was further distorted as the cell wall was inclined to degrade (Figure 1H). Both protein and lipid bodies exhibited damage without new formation under calcium-deficient conditions. Moreover, as the endomembrane systems tended to decompose, the organelles showed a disordered arrangement inside the embryo cells. By contrast, massive lipid bodies continued to form around many vacuole protein bodies in an orderly manner in embryo cells under calcium-sufficient conditions (Figure 1G). Therefore, calcium deficiency caused irregular embryo development at early stages, especially the membrane systems, which impaired the accumulation of reserve substances including starch, proteins and lipids, leading to the abortion of seeds.

### Global RNA-seq Delineated Genome-Wide Transcriptome Changes in Response to Calcium Deficiency

To investigate responsive genes to calcium deficiency in peanut embryos, RNA-seq libraries for calcium deficiency and sufficiency at 15, 20 and 30 DAP were constructed and the global gene expression profiles surveyed using the Illumina HiSeq™ 2500 platform. After removal of adaptor sequences and low-quality reads, a total of ∼20–26 million clean reads for each sample were produced, comprising 100,743,125,153 nucleotides (100.74 Gb) with ∼90% Q30 and ∼46% GC content (Supplementary Table 1). After alignment with the peanut reference genomes (Zhuang et al., 2019), a high proportion of reads (∼86–93%) were mapped to the genomes. Among them, ∼69–82% were mapped uniquely to one location and more than 80% to the exons (Supplementary Figure 1). The transcriptional sufficiency of the genes was quantified using Cufflinks and measured as Fragments Per Kilobase per Million mapped reads (FPKM). As a result, 60,011 transcripts were identified. Accordingly, we were convinced that the obtained sequencing qualities were sufficiently high to sustain further analysis.

**FIGURE 2:**
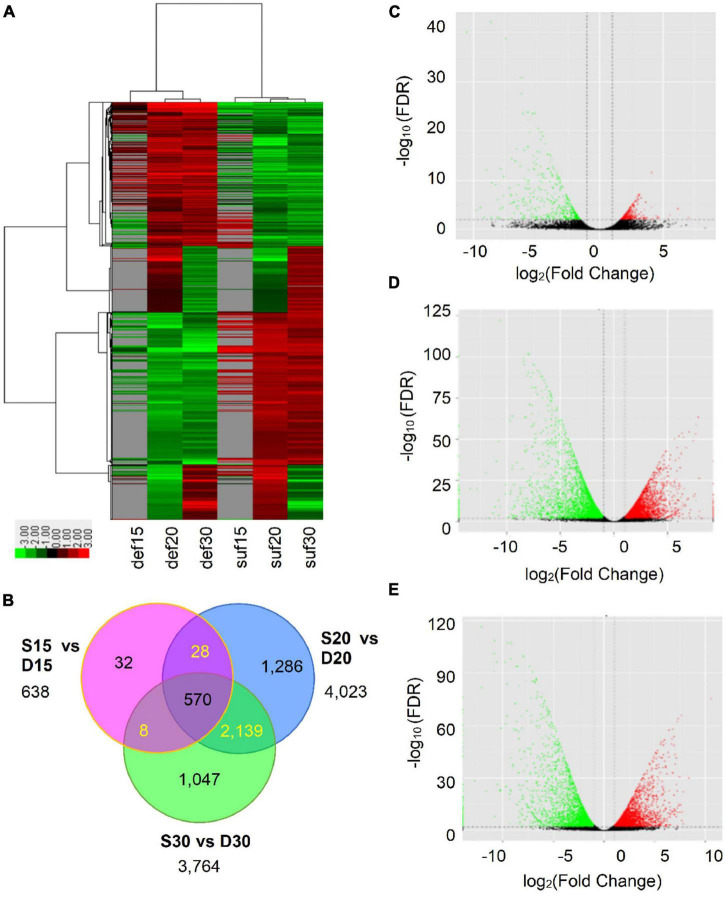
Transcriptional changes in peanut embryos responded to calcium deficiency. **(A)** Expression profiles of the DEGs response to calcium deficiency were analyzed and clustered by average linkage method. Expression level was calculated using log_2_ (FPKM + 1). Red indicates up-regulated genes, green indicates down-regulated genes and gray indicates no expression data. **(B)** The distribution of differentially expressed transcripts. **(C)** The volcano plot of DEGs at 15 DAP. **(D)** The volcano plot of DEGs at 20 DAP. **(E)** The volcano plot of DEGs at 30 DAP. FDR in panels **(C–E)** means false discovery rate.

To identify calcium deficiency-responsive genes that regulate embryo development in peanut, the normalized expression levels of all genes were subsequently analyzed for their expression patterns. A total of 4,540 DEGs were identified, of which 638, 4,023, and 3,764 DEGs were obtained at 15, 20 and 30 DAP (Figure 2A and Supplementary Table 2). Among them, a total of 337, 2,028 and 1,898 DEGs were up-regulated and 301, 1,995 and 1,866 DEGs down-regulated at 15, 20 and 30 DAP, respectively (Figures 2C–E and Supplementary Table 2). Additionally, 570 DEGs showed differential expression at the three stages. Among these DEGs, 319 were up-regulated and 251 down-regulated (Figures 2B, 4E and Supplementary Table 2). The differentially expressed genes reflected the broad molecular changes associated with peanut embryo abortion under calcium deficiency conditions.

### Annotation and KEGG Pathway Analysis for Differentially Expressed Genes

**FIGURE 3:**
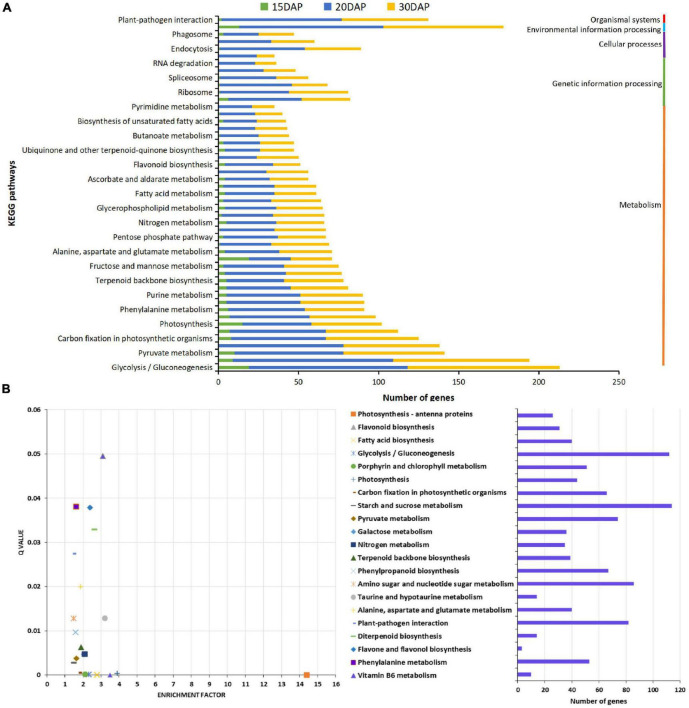
KEGG pathways analysis for differentially expressed genes. **(A)** Statistical analysis of KEGG pathways for DEGs; **(B)** KEGG pathways enrichment analysis of DEGs.

To further explore the biological function of DEGs during peanut embryo abortion under calcium deficiency, the DEGs were annotated based on the different databases. A total of 244, 130 and 176 enriched GO terms in biological process, cellular component and molecular function, respectively, were identified among the DEGs. GO term enrichment analysis indicated that signal transduction, biosynthetic processes of plant hormones, and responses to biotic and abiotic stimulus were significantly enriched (Supplementary Figures 2, 4 and Supplementary Tables 3, 5). Furthermore, KEGG pathway analysis indicated DEGs were assigned to 68, 113, and 113 pathways involved in peanut embryo abortion under calcium deficiency at 15, 20, and 30 DAP, respectively. It is worth noting that plant hormone signal transduction and plant-pathogen interaction pathways were also identified as significantly enriched (Figure 3A). Most DEGs participating metabolism, plant hormone levels and the plant-pathogen interaction presumably play vital roles in peanut embryo abortion under calcium deficiency. Among them, 21 pathways were significantly enriched (Q value < 0.05), including photosynthesis-antenna proteins, photosynthesis, flavonoid biosynthesis and fatty acid biosynthesis (Figure 3B and Supplementary Table 6). These pathways clearly conformed to the morphological variances of embryo development and differences in reverse synthesis and accumulation, as observed based on the number and size of starches, protein bodies, and lipid bodies between calcium sufficiency and deficiency conditions (Figure 1).

### Differential Expression of Differentially Expressed Genes Related to Embryo Abortion

**FIGURE 4:**
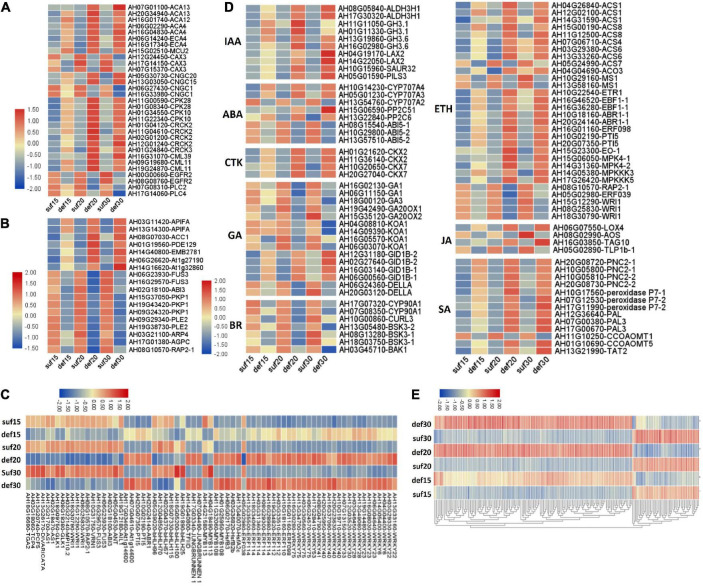
Relative expression changes of important differential expression genes and microarray validation of expression profiles of identified DEGs under deficiency calcium. **(A)** Calcium signaling pathway-related genes. **(B)** Embryo development related genes. **(C)** Transcription factors. **(D)** Plant hormone related genes. IAA, indole-3-acetic acid; ABA, abscisic acid; CTK, cytokinin; GA, gibberellin; BR, brassinosteroid; ETH, ethylene; JA, jasmonic acid; SA, salicylic acid. **(E)** Parts of common DEGs at the three stages. The expression level was calculated using log_2_ (FPKM + 1).

Given the importance of calcium in peanut growth and development, we were first prompted to focus on calcium signaling pathway-related genes for revealing the relationship between calcium and embryo development. The expression levels of many genes involved in calcium signaling pathway were significantly altered, presumably in association with embryo abortion under calcium deficiency. A group of genes controlling Ca^2+^ efflux and influx presented expression changes (Figure 4A and Supplementary Table 7). Among them, eight DEGs encoding Ca^2+^ transporting ATPases (ACA13, ACA12, ACA4 and ECA4) and one DEG encoding the mitochondrial calcium uniporter protein (MCU2) were up-regulated under calcium deficiency. Three DEGs encoding vacuolar cation/proton exchanger 3 (CAX3) showed an apparent down-regulation. Additionally, there was a difference in the accumulation of transcript levels of four DEGs encoding the cyclic nucleotide-gated ion channel (CNGC), of which *CNGC15* and *CNGC20* were up-regulated and *CNGC1* was down-regulated. In addition, epidermal growth factor receptor (EGFR) and phosphatidylinositol phospholipase C (PI-PLC, PLC2 and PLC4) were down-regulated. Accordingly, it was predicted that calcium deficiency might induce embryo abortion through expression changes in these genes regulating cytoplasmic Ca^2+^ homeostasis. In contrast, calcium signal components, such as calcium-dependent protein kinases (CPK28, CPK10), calmodulin-binding receptor-like cytoplasmic kinases (CRCK2, CRCK3) and calmodulin-like proteins (CML11, CML39), were up-regulated. It is suggested the Ca^2+^ homeostasis was altered in the cytoplasm under calcium deficiency, resulting in defects in embryo development as a consequence of embryo abortion.

### The Expression of Embryo Developmental Genes Altered in Response to Ca^2+^ Deficiency

Embryo developmental genes among the DEGs according to the annotation results was then investigated. It was reported that 481 genes related to seed development in Arabidopsis were disrupted by a loss-of-function mutation exhibiting a seed phenotype (Meinke et al., 2008). To identify peanut embryo developmental genes, a blast analysis of our DEGs against these 481 genes was performed. Among all the DEGs, four were predicted to encode plastidial pyruvate kinase 2 (pPK2), which showed a high similarity to PKP1 in Arabidopsis and were down-regulated in peanut embryos under calcium deficiency (Figure 4B and Supplementary Table 7). Two DEGs encoding the B3 domain-containing transcription factor FUS3 and one DEG encoding ABI3 displayed significant down-regulation in the three developmental stages (Figure 4B and Supplementary Table 7). Two DEGs were predicted to encode dihydrolipoyllysine-residue acetyltransferase component 4 of the pyruvate dehydrogenase complex (LTA2), which shared high similarity to PLE2 in Arabidopsis, as well as a down-regulation in peanut embryos under calcium-deficient conditions (Figure 4B and Supplementary Table 7). One DEG was predicted to encode the ethylene-responsive transcription factor RAP2-1, which was significantly down-regulated under calcium deficiency (Figure 4B and Supplementary Table 7). Furthermore, eight DEGs were significantly up-regulated under calcium-deficient conditions (Figure 4B and Supplementary Table 7). Among the up-regulated DEGs, AH14G40800 was suggested to encode the transcription initiation factor TFIID subunit 6, which exhibits high similarity to EMB2781 in Arabidopsis. Two DEGs encoding the leucine-rich repeat protein kinase family protein and one for the glycosyl hydrolase superfamily protein showed a remarkable up-regulation. These DEGs might also play important roles in peanut embryo abortion under calcium deficiency conditions.

### The Expression of Genes Involved in Hormone Biosynthesis and Signal Transduction

A series of genes related to plant hormones biosynthesis and signal transduction were differentially expressed in calcium deficiency-treated peanut embryos (Figure 4C and Supplementary Table 7). Aldehyde dehydrogenase family 3 member H1 (ALDH3H1) participating in tryptophan metabolism and in indole-3-acetic acid (IAA) biosynthesis were up regulated. The indole-3-acetic acid-amido synthetase GH3.1 and GH3.6 were also significantly up-regulated. Auxin transporter protein1 (AUX1), protein PIN-LIKES 3 (PILS3) and auxin-responsive protein SAUR32 were significantly up-regulated under deficient calcium stress. Cytokinin dehydrogenase (CRX), including CKX2 and CKX7, were significantly up-regulated under calcium deficiency. For gibberellin, the ent-copalyl diphosphate synthases GA1 and GA3, gibberellin 20 oxidases GA20OX1 and GA20OX2, and ent-kaurenoic acid oxidase 1 (KAO1) were down-regulated. Surprisingly, gibberellin receptor GID1B was up-regulated and DELLA protein GAI down-regulated. In terms of the brassinosteroid signaling transduction pathway, the brassinosteroid LRR receptor kinase CURL3 and protein kinase protein with the tetratricopeptide repeat domain (BSK3) and BRASSINOSTEROID INSENSITIVE 1-associated receptor kinase 1 (BAK1) were all significantly down-regulated.

**FIGURE 5:**
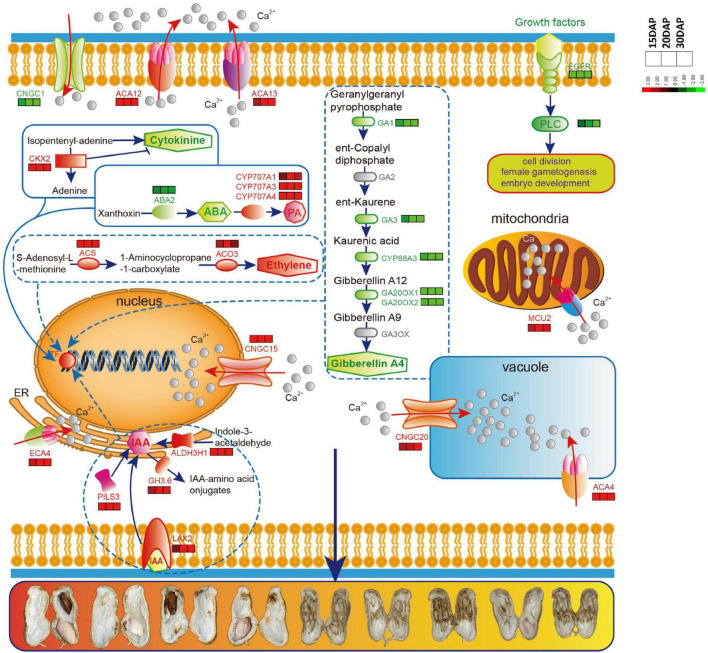
The predicted schematic diagram of calcium signaling pathway and hormone biosynthesis related genes in peanut embryo cells and their subcellular localization. Red color indicates up-regulated genes, green indicates down-regulated genes. Calcium-transporting ATPases and Cyclic nucleotide-gated ion channels (CNGC) are localized in different intracellular organelles and have high capacity Ca^2+^ flow across membranes. ACA12 and ACA13 are present in the plasma membrane, ACA4 in the vacuole and ECA4 in endoplasmic reticulum (ER). CNGC1 is in the plasma membrane, CNGC15 in nucleus and CNGC20 in vacuole membrane. EGFR, epidermal growth factor receptor; PLC, phosphoinositide phospholipase C; MCU2, calcium uniporter protein 2, mitochondrial; GA1, Ent-copalyl diphosphate synthase; GA3, Ent-kaurene oxidase; GA20OX1, Gibberellin 20 oxidase 1; GA20OX2, Gibberellin 20 oxidase 2; CKX2, Cytokinin dehydrogenase 2; ABA2, Xanthoxin dehydrogenase; CYP707A1, Abscisic acid 8′-hydroxylase 1; CYP707A3, Abscisic acid 8′-hydroxylase3; CYP707A4, Abscisic acid 8′-hydroxylase 4, ACS, 1-aminocyclopropane-1-carboxylate synthase; ACO3, PILS3, protein PIN-LIKES 3; GH3.6, Indole-3-acetic acid-amido synthetase GH3.6; ALDH3H1, Aldehyde dehydrogenase family 3 member H1.

The abscisic acid 8′-hydroxylase genes (*CYP707A1*, *CYP707A3*, *CYP707A4*) were up-regulated in calcium deficiency peanut embryos (Figure 5). Moreover, the xanthoxin dehydrogenase gene *ABA2* and zeaxanthin epoxidase gene *ABA1* involved in the biosynthesis of abscisic acid were down-regulated. The ABA receptor PYL showed expression changes with *PYL9* down-regulation and *PYL4* and *PYL11* up-regulation. Additionally, two protein phosphatase 2C (PP2C) were also up-regulated. ABSCISIC ACID-INSENSITIVE 5 (ABI5) was down regulated. The expression changes in ABA signal transduction pathway-related genes should be responsible for embryo abortion under calcium deficiency conditions. A series of genes involved in ethylene biosynthesis and signal transduction exhibited altered expression. For example, 1-aminocyclopropane-1-carboxylate synthase 1 (ACS1) and 1-aminocyclopropane-1-carboxylate oxidase 3 (ACO3) related to ethylene biosynthesis were up-regulated. Ethylene receptor 1 ETR1, ethylene-responsive transcription factor ABR1 and ERF098 and the pathogenesis-related gene transcriptional activator PTI5 were all up-regulated. Mitogen-activated protein kinase 4 (MPK4), mitogen-activated protein kinase kinase kinase 3 (MAPKK3) and mitogen-activated protein kinase kinase kinase 5 (MAPKK5) were also up-regulated. However, several other ethylene-responsive transcription factors, including RAP2-1, ERF039 and WRI1, were down-regulated. Moreover, the TGACG-sequence-specific DNA-binding protein TGA10 and thaumatin-like protein TLP1b were up-regulated and down-regulated, respectively, in the jasmonic acid signaling pathway. Both peroxidases, including cationic peroxidase 2 (PNC2) and peroxidase P7-1, as well as phenylalanine ammonia-lyase (PAL), were up-regulated in the salicylic acid signaling pathway. Briefly, the altered transcript levels of the above genes in peanut embryos under calcium deficiency condition should change the plant hormone levels and affect signal transduction.

### Different Transcription Factor Families Showed Distinct Expression Changes

A total of 24 WRKY transcription factors, 10 ethylene-responsive transcription factors (ERFs) containing 5 ERF families, 3 heat stress transcription factors and 4 MYB transcription factors, were all up-regulated in response to low calcium (Figure 4D and Supplementary Table 7). Additionally, two pathogenesis-related gene transcriptional activators (PTI5) and two transcription factors JUNGBRUNNEN 1 were also up-regulated under calcium-deficient conditions.

The down-regulated transcription factors under deficient calcium conditions included 6 bHLH transcription factors, 5 ethylene-responsive transcription factors, 5 B3 domain-containing transcription factors and 3 AP2-like ethylene-responsive transcription factors. Importantly, two transcription factors related to embryogenesis or ovule development were significantly down-regulated, including the transcription factor *TCP4* encoding protein MATERNAL EFFECT EMBRYO ARREST 35 (MEE35) and AP2-like ethylene-responsive transcription factor *ANT*. In addition, several transcription factors related to cell differentiation were also down-regulated, such as *MYB113*, *AS1* and GATA transcription factor 21. Several other transcription factors that participate in development or defense responses, such as *GLK1* (related to plastid development and resistance), *DIVARICATA*, *PCF5* and *TGA2*, were also down-regulated.

### Validation for Expression Profiles of the Identified Differentially Expressed Genes

**FIGURE 6:**
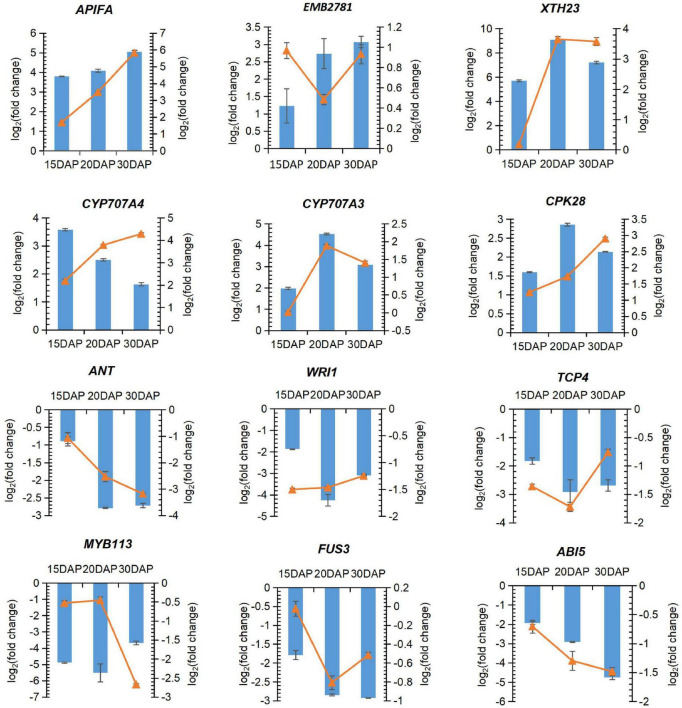
Quantitative RT-PCR validation of selected DEGs under calcium deficiency and sufficiency in peanut embryo at 15, 20 and 30 DAP. Blue bar represents relative expression level changes calculated by the FPKM using RNA-seq. Orange line with indicates relative expression level changes determined by qRT-PCR analysis.

**FIGURE 7:**
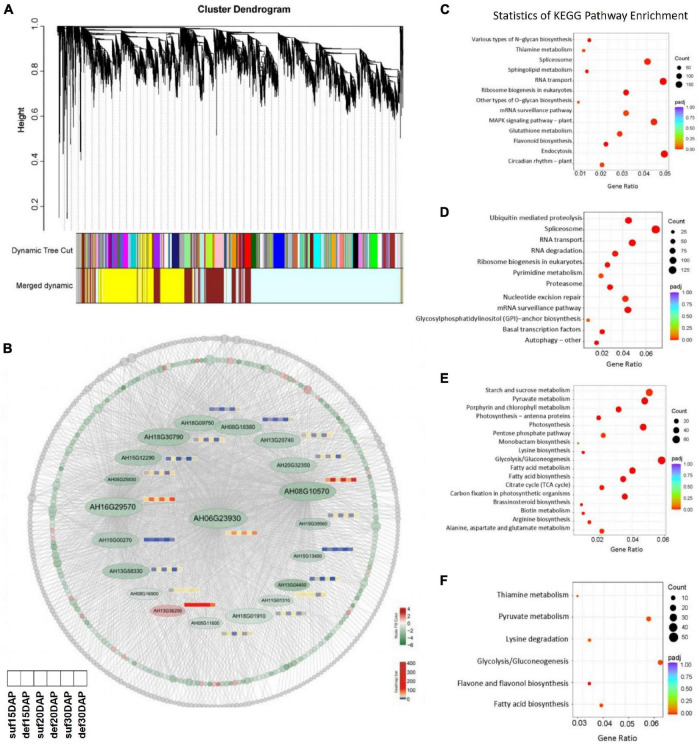
WGCNA of the genes under calcium deficiency and sufficiency in peanut embryo at 15, 20 and 30 DAP. **(A)** Hierarchical clustering tree indicates 16 modules of co-expressed genes identified by WGCNA. **(B)** Network of the hub gene *AhFUS3* (AH06G23930) and related genes with an edge weight of > 0.15 from the navajowhite2 module. Each circle represents one gene and red color indicates up-regulated genes, green indicates down-regulated genes, gray color represents the genes with no significant differences under calcium deficiency and sufficiency conditions. The expression profiles for important transcription factors in the navajowhite2 module under calcium deficiency and sufficiency conditions at the stage of 15, 20 and 30 DAP were analyzed. **(C–F)** KEGG analysis of genes in the darkolivegreen2, firebrick3, navajowhite, navajowhite2 modules.

A total of 12 important candidate DEGs were then selected for the quantitative RT-PCR assays to confirm the differential expression of the important DEGs screened from the RNA-Seq analysis. The expression levels of the DEGs fit well with those determined by RNA-Seq analysis (Figure 6). Two DEGs encoding apoptosis-inducing factor homolog A (APIFA) showed sharp increases in calcium deficiency-treated embryos at 15, 20 and 30 DAP (Figures 4B, 7). APIFA presumably might play a pivotal role in peanut embryo abortion under calcium deficiency. The relative expression changes in two transcription factors (*TCP4* and *ANT*) related to embryogenesis or ovule development were also validated by qRT-PCR suggesting that they were down-regulated in the calcium deficiency-treated embryos, consistent with the RNA-seq (Figures 6, 4D). The down-regulation of these two transcription factors might play a key role in peanut embryo development barriers under conditions of calcium deficiency.

### WGCNA Identified Correlated Gene-Modules and Highly Connected Hub Genes

WGCNA was performed to construct the co-expression networks across all samples and 16 distinct gene co-expression modules defined using different color codes were identified (Figure 7A). The genes with high correlation were clustered into one module, and genes within the same module are co-expressed. KEGG pathway enrichment analysis showed that the pathways related to MAPK signaling pathway was significantly enriched in the darkolivegreen2 module, ubiquitin mediated proteolysis pathway was significantly enriched in the firebrick3 module, reserve substance biosynthesis and metabolism such as fatty acid, starch and sucrose, lysine and arginine was significantly enriched in the navajowhite and navajowhite2 modules (Figures 7A,C–F and Supplementary Table 8). Cytoscape 3.9.0 was used to construct the co-expression network of hub gene *FUS3* (AH06G23930), which had the highest connectivity among the navajowhite2 module and plays important roles in embryo development (Figure 7B). Interestingly, several other transcription factors such as *TCP4*, *GLK1*, *ABI3*, *bHLH115*, *MYC2*, etc., were also included in this module and down regulated under calcium deficiency (Figures 4C, 6 and Supplementary Tables 9, 10), which had been identified to be involved in embryogenesis or ovule development. Thus these genes in navajowhite2 module may play important roles in peanut embryo development.

## Discussion

### Deficient Ca^2+^ Impaired Developing Embryo Cells and Reversed Accumulation, Leading to Embryo Abortion

Peanut grown in calcium-deficient soil generates a high degree of empty pods, showing special sensitivity to calcium level in the soil (Jain et al., 2011). Deficiency calcium severely impaired the normal development of embryo cells, causing the endomembrane system disintegrated, the cell wall to remain thin and later degrade, and the vacuoles to fail compartmentation in early stages and thus leading to reserve accumulation (Figure 1). The abnormal embryo cell growth was clearly the first step toward embryo abortion. As a result, the accumulation of reserve substances, including starch granules, protein and lipid bodies, were all seriously impaired and dissolved (Figure 1). Thereby, deficient calcium weakened early embryo cell development, abrogating reverse synthesis and accumulation and thus leading to embryo abortion.

### Gene Expression Profile Changes Under Calcium Deficiency Were Consistent With Embryo Cell Development and Reverses Biosynthesis

To elucidate the cellular events, a large number of DEGs were identified involved in various pathways (Figure 3). Diverse important biological processes, such as signal transduction, biosynthetic processes of plant hormones, and responses to biotic and abiotic stimuli were significantly enriched (Supplementary Figures 2, 4 and Supplementary Tables 3–5). Specifically, many genes involved in carbohydrate metabolic pathways such as starch and sugar metabolism, glycolysis and gluconeogenesis, and citrate cycle pathways, for example, the alpha-amylase gene *AMY1.1*, were up-regulated in response to calcium deficiency (Figure 3; Yu et al., 2018). These phenomena explain the reduced number of plastids in early embryo cells and greatly reduced starch accumulation size (Figure 1). Genes related to fatty acid biosynthesis and metabolism and glycerolipid and glycerophospholipid metabolisms pathways, such as *TGL1* (Fan et al., 2017), *WRI1* (Chen et al., 2018), *LOX1.5* (Lõpez et al., 2011), lipid-transfer protein 1 (LTP1) and peroxygenase (SOP1), regulating fatty acid biosynthesis, were down-regulated. These results were consistent with the decreased accumulation and distorted morphology of lipid bodies (Figure 1). Many genes, such as amino acid permease 7 (AAP7), an amino acid-proton symporter and a stereospecific transporter with broad specificity for neutral amino acids in amino acid metabolism and protein synthesis and processing, such as glycine, alanine, aspartate, glutamate, and phenylalanine metabolism, processing, and amino sugar and nucleotide sugar metabolism pathways, were down-regulated, impairing protein synthesis, as observed in the protein bodies behavior in calcium-deficient embryo cells (Figure 1).

It was also detected several up-regulation for cell wall degradation-related genes but down-regulation of cell wall biogenesis and organization-related genes (Supplementary Table 2). XTH, participating in cell wall biogenesis and construction (Miedes et al., 2013), was up-regulated under calcium deficiency. PME, related to the modification of cell walls *via* demethylesterification of cell wall pectin, was also up-regulated. The up-regulation of XTH and PME could probably lead to a loss of the cell wall and a distorted cell morphology and structure, consistent with the TEM observation (Figure 1). Therefore, the transcriptome changes under calcium deficiency were likely responsible for the distorted cell morphology and structure, abated embryonic cell growth and hindered accumulation of lipid and protein bodies, ultimately leading to embryo abortion.

### Genes That Function in Calcium Signaling Pathways Abrogated Embryo Cell Development Under Low Ca^2+^

Cytoplasmic Ca^2+^ signals have been reported to play vital roles in the activation of programmed cell death (PCD) (Ma and Berkowitz, 2007). Cytoplasmic Ca^2+^ homeostasis is essential to mediate plant growth and development, as well as adaptive responses to a broad range of abiotic and biotic stresses (Clapham, 2007). In the present study, many calcium-related genes were changed under calcium deficiency. Genes related to Ca^2+^ influx, such as *CNGC1* and *CNGC17*, were down-regulated, and genes controlling Ca^2+^ efflux, such as *ACA4*, *ACA12*, *ACA13*, *ECA4* and *MCU2*, were up-regulated (Figure 5). *CNGC1* has been confirmed to relate Ca^2+^ uptake and root development in Arabidopsis (Ma et al., 2006). *CNGC17* plays important roles in phytosulfokine (PSK) peptide-induced cell expansion (Ladwig et al., 2015). Additionally, the expression level of *CNGC15* in the present study was increased, resulting in a flow of Ca^2+^ flow from the cytoplasm to the nucleus (Figure 5). *Mt-CNGC15a/b/c* have been found to be responsible for nuclear Ca^2+^ spiking in *Medicago truncatula* (Charpentier et al., 2016). *CNGC20* is also up-regulated and involved in Ca^2+^ oscillations in the nucleus, playing essential roles in the establishment of both rhizobial and mycorrhizal symbioses in roots (Capoen et al., 2011). Obviously, the above genes control cytoplasmic Ca^2+^ homeostasis and are intimately involved in plant development. Notwithstanding, little is currently known about their effects on embryo development. In addition, *EGFR*, *PLC2* and *PLC4* were down-regulated. Accumulating evidence places PI-PLC enzymes at the center of plant innate immunity, which can regulate the increased cytoplasmic levels of free Ca^2+^, lowering the intercellular pH and the oxidative burst (Abd-El-Haliem and Joosten, 2017). Hence, reduced expression levels of PI-PLC lead to a decrease in cytoplasmic levels of free Ca^2+^. Importantly, PLC2 is essentially involved in reproductive and embryonic development, presumably by regulating mitosis and/or formation of the cell division plane (Di Fino et al., 2017). Disruption of PLC2 leads to sterility, indicating a significant role of PLC2 in reproductive development (Li et al., 2015). Especially important is the observation of elevated auxin levels in plc2 floral tissues, suggesting that the infertility of plc2 plants may be associated with increased auxin concentrations in reproductive organs (Li et al., 2015). Therefore, calcium deficiency induced embryo abortion associated with expression changes in these genes by regulating cytoplasmic Ca^2+^ homeostasis.

During calcium signal transduction, Ca^2+^-binding proteins or Ca^2+^ sensors decode the stimulus-specific Ca^2+^ signals into downstream responses. In the present study, many calcium signal components, such as CPK28, CPK10, CRCK2, CRCK3, CML11 and CML39, were up-regulated. As calcium sensors, calcium-dependent protein kinases (CDPKs) play important roles in the regulation of plant growth and development, as well as responses to biotic and abiotic stresses. In Arabidopsis CPK28 controls stem elongation and vascular development (Matschi et al., 2013). CPK28 targets methionine adenosyltransferase (MAT), an essential enzyme in ethylene biosynthesis. cpk28 mutants display short hypocotyls and ectopic lignification caused by ethylene overproduction (Jin et al., 2017). Furthermore, CPK28 also regulates development by balancing JA and GA levels (Matschi et al., 2015). When plants are exposed to microbial infection, the primary responses mediated by surface-localized immune receptors are to increase cytosolic calcium (Ca^2+^) levels followed by a burst of apoplastic reactive oxygen species (ROS). CPK28 negatively regulates the BIK1-mediated PAMP-induced calcium burst (Monaghan et al., 2015). Hence, CPK28 is an important regulatory component related to plant growth and development, associated with changes in phytohormones and cytosolic Ca^2+^ levels. In the present study, CPK28 was significantly up-regulated under conditions of calcium deficiency, concurring with aborted embryos and decreases in cytosolic Ca^2+^ levels. CPK10 participates in ABA- and Ca^2+^-mediated regulation of stomatal movements in response to drought stress (Zou et al., 2010). Notwithstanding, the functions of CPK10 in seed/embryo development have remained unclear. Here, CPK10 was up-regulated under calcium deficiency, implying its important roles in peanut embryo development. CML39 belonging to extended families of unique Ca^2+^ sensors, is involved in light signal transduction and early seedling establishment (Bender et al., 2013). CML11 participated in the defense response against *Verticillium dahliae* infection in upland cotton (Cheng et al., 2016). However, the functions of CML in seed/embryo development have not been clarified. In addition, CRCK2 and CRCK3 are also up-regulated. Disruption of CRCK2 induces defects in male gametophyte development (Boavida et al., 2009). The up-regulation of CRCKs might suggest that peanut plants have an adaptive reaction to conditions of calcium deficiency. *De novo* transcriptome sequencing has revealed potential mechanisms of seed abortion in dove tree and shown that calcium might be an important second messenger in the regulation of abortion (Li et al., 2016). A number of calcium-related genes, including CML and ACA, are up-regulated in aborted seeds (Li et al., 2016). The expression of three Ca^2+^-transporting ATPase genes were lower in peanut kernels under the Ca^2+^ sufficient treatment (Yang et al., 2020). All the above results are consistent with ours.

Accordingly, a model was proposed to generalize Ca^2+^ homeostasis in embryo cells under calcium deficiency conditions (Figure 5). Calcium deficiency first decreased the Ca^2+^ level in the apoplast and tissue. Subsequently, it altered the transcriptome through the expression of genes encoding Ca^2+^ influx and efflux transporters, allowing free Ca^2+^ in the cytoplasm and extracellular space to enter intracellular organelles, including the vacuole, mitochondria, endoplasmic reticulum and nucleus, leading to a decline in cytoplasmic free Ca^2+^ levels. These changes clearly disrupt cytoplasmic Ca^2+^ homeostasis, mediate the hormone metabolic balance, alter gene expression, and ultimately lead to peanut embryo abortion under calcium deficiency conditions through hormone signaling and transcriptome changes (Figure 5).

### Plant Hormone Signaling Plays Key Roles in Peanut Embryo Abortion

A series of genes related to plant hormone biosynthesis and signal transduction were differentially expressed in calcium-deficient peanut embryos (Figure 3). The endogenous IAA content is significantly increased in aerial pods with embryo abortion (Chen et al., 2013). IAA content increased in peanut kernels under Ca^2+^ deficiency (Yang et al., 2020). In our study, ALDH3H1, participating in tryptophan metabolism and the key enzyme in IAA biosynthesis, was up-regulated. IAA synthetase GH3.1 and GH3.6 were also up-regulated, which catalyze the synthesis of IAA-amino acid conjugates, providing a mechanism for plants to cope with the presence of excess auxin (Staswick et al., 2005) in embryo cells and this result was consistent with the previous reports (Yang et al., 2020). Auxin transporter protein (AUX1/LAX2) was significantly up-regulated under deficient calcium stress, presumably leading to an increase in IAA content. PILS3, required for auxin-dependent regulation of plant growth by determining the cellular sensitivity to auxin, was also up-regulated. In Arabidopsis, ectopic expression of PILS3 resulted in dwarfed and/or bushy plants showing severe defects in flower development leading to sterility (Barbez et al., 2012). The related genes in the auxin signaling transduction pathway, including the auxin-responsive protein SAUR32, were also markedly up-regulated. Over-expression of SAUR32 leads to reduce hypocotyl elongation in Arabidopsis (Park et al., 2007). It is possible that the up-regulation of SAUR32 should result in some defects in peanut embryo cell growth.

Cytokinin dehydrogenase 2 (CKX3) and CKX7 were significantly up-regulated in peanut embryo under calcium deficiency. CKX is a key negative regulator controlling endogenous cytokinin in plants (Zhao et al., 2015). Thereby, this change could lead to a decrease in endogenous cytokinin, which might affect seed development and ultimately lead to embryo abortion. However, the previous study showed that two genes encoding cytokinin dehydrogenase were down-regulated in peanut kernels under Ca^2+^ deficient conditions (Yang et al., 2020), which is contrary to our findings. Therefore, cytokinin content in peanut seeds under calcium deficiency and sufficiency conditions should be assayed in the future study.

The level of endogenous abscisic acid has been reported to function as a positive regulator during plant embryo development (González-Guzmán et al., 2002). *CYP707As* encode abscisic acid (ABA) 8′-hydroxylases, key enzymes in ABA catabolism. *CYP707A1*, *CYP707A3*, and *CYP707A4* were up-regulated in calcium-deficient peanut embryos, leading to a decrease in the endogenous ABA level. Previous studies have demonstrated that overexpression of *AhCYP707A4* in *Nicotiana benthamiana* decreased ABA content with high numbers of aborted embryos, small pods and fewer seeds, confirming role in the regulation of Ca^2+^ deficiency-induced embryo abortion *via* ABA-mediated apoptosis (Chen et al., 2016). Additionally, *ABA2* and *ABA1*, involving in the biosynthesis of ABA, were down-regulated. ABA2 catalyzes the conversion of xanthoxin to abscisic aldehyde, a key step in ABA biosynthesis (González-Guzmán et al., 2002). The up-regulation of *CYP707As* and down-regulation of *ABA2* and *ABA1* might thus decrease the ABA level under calcium deficiency conditions, affecting embryo development. It was reported that abscisic acid 8′-hydroxylase gene was up-regulated in peanut kernels under Ca^2+^ deficiency, which is also consistent with our results (Yang et al., 2020).

Several key enzymes involved in gibberellin biosynthesis were down-regulated under deficient calcium conditions: GA1 and GA3 (Sun and Kamiya, 1994), GA20OX1 and GA20OX2 (Rieu et al., 2008), and KAO1 (Helliwell et al., 2001). Gibberellin 20 oxidase gene were down-regulated in peanut kernels under Ca^2+^ deficiency, which is also consistent with our results (Yang et al., 2020). The down-regulation of these genes decrease the GA level in calcium deficiency-treated embryos, affecting embryo development. Surprisingly, the gibberellin receptor GID1B was up-regulated and the DELLA protein GAI down-regulated. GID1B is expressed in ovules as a dominant GA receptor at low GA concentrations during germination (Gallego-Giraldo et al., 2014). GID1B targets DELLA proteins, the repressors of GA-induced growth, for degradation *via* the proteasome as the feedback mechanism caused by low levels of GAs in calcium-deficient embryos.

Several genes in the brassinosteroid signaling pathway were down-regulated, including *CYP90A1*, *CURL3*, *BSK3* and *BAK1*. *CYP90A1* (*CPD*), a cytochrome P450 gene encoding steroid hydroxylase, is a BR-specific biosynthesis gene and controls cell elongation (Tanaka et al., 2005). Brassinosteroids could rescue the phenotype of the cpd mutant (Szekeres et al., 1996). CURL3 responds to BR, and the curl-3 mutant shows reduced fertility, strikingly similar to the Arabidopsis mutant brassinosteroid insensitive 1 (bri1) (Koka et al., 2000). BSK3 is a positive regulator of BR signaling (Zhang et al., 2016). BAK1 and BKK1 regulate BR-dependent growth and BR-independent cell-death pathways, and bak1 bkk1 double mutants exhibit a seedling-lethality phenotype (He et al., 2007). It was also found that *CYP90A1* was down-regulated under calcium deficiency, which should decrease BR levels in the embryo and affect the expression of downstream gene, such as *CURL3*, *BSK3* and *BAK1*, ultimately affecting normal development.

In addition, many genes involved in ethylene biosynthesis and signal transduction showed different expression levels under low calcium conditions. For example, *ACS1* and *ACO3*, which are related to ethylene biosynthesis, were up-regulated, possibly leading to an increase in ethylene levels. Accordingly, ethylene receptor (ETR), EIN3-binding F-box protein 1 (EBF1), and ethylene-responsive transcription factors (ABR1 and ERF098) were also up-regulated. The increased expression of these genes might promote advanced embryo maturity and halt continuing development, as supported by the observation that peanuts growing in calcium deficiency soil produced many more geminated seeds.

In addition, some important genes in the jasmonic acid and salicylic acid signal transduction pathways were also significantly changed. Thus, peanuts growing in calcium-deficient conditions activated a series of gene expression changes and cross-talk among various hormones.

Overall, peanut embryo development is associated and regulated by various hormones. However, the mechanisms that regulate the crosstalk among these plant hormones are still not well understood. Furthermore, changes in the cytoplasmic calcium concentration have been demonstrated to function as a secondary signal messenger for most plant hormone pathways (Fortes et al., 2015). Recently, a growing number of studies have shown that the calcium signal transduction pathway is involved in plant hormone pathways. CPK28 plays a vital role in balancing JA and GA in Arabidopsis development (Matschi et al., 2015) and affects ethylene biosynthesis (Jin et al., 2017). CPK4 is related to the modulation of ABA signaling (Wang et al., 2017). CML20 negatively regulates ABA-induced stomatal movement in Arabidopsis (Wu X. et al., 2017). StCDPK3 is involved in the cross-talk between ABA and GA signaling at the onset of potato tuber development (Grandellis et al., 2016). In brief, peanut seed development may be regulated by the collaboration of the Ca^2+^ signal transduction pathway and its involvement in hormone regulation pathways. In a word, a predicted schematic diagram was proposed to generalize calcium signaling pathway and hormone biosynthesis related genes in peanut embryo cells according to our and previous study (Figure 5). The predicted schematic diagram of calcium signaling pathway and hormone biosynthesis related genes in peanut embryo cells and their subcellular localization. More research is needed to delve into the underlying molecular mechanisms for peanut abortion under calcium deficiency conditions.

### Ca^2+^ Deficiency Affects Other Ion Transport and Nutrient Absorption in Peanut Embryo

Previous study indicated that Ca^2+^ deficiency can affect several other ions transport (zinc, aluminum, sodium, potassium, iron, and magnesium) and nutrient absorption (nitrate and phosphate) (Yang et al., 2020). Here we found that zinc transporter 5 was up-regulated under calcium deficiency (Supplementary Table 2). Sodium/hydrogen exchanger 4 (NHX4), sodium/metabolite cotransporter BASS1 (T30F21.11) and sodium-dependent phosphate transport protein 1 (T27A16.25) were down regulated (Supplementary Table 2). Two DEGs encoding potassium transporter 8 (T9L3_180) were down regulated (Supplementary Table 2). However, DEGs encoding potassium transporter 5 (T9E8.160), potassium transporter 12 (T13D8.5) and potassium channel SKOR (SKOR) were up regulated (Supplementary Table 2). Several DEGs related to magnesium transport including magnesium transporter MRS2-1, MRS2-3, MRS2-4, MRS2-11 were also found (Supplementary Table 2). Nitrogen, phosphorus and potassium are essential elements for plant growth and development. In this study, the expression levels of fourteen NRT1/PTR family protein genes were changed with four genes down-regulated and ten genes up-regulated under Ca^2+^ deficiency conditions (Supplementary Table 2). In addition, one DEG encoding high-affinity nitrate transporter 3.1 (At5g50200) and one DEG encoding peptide/nitrate transporter (ZIFL2) were up regulated under Ca^2+^ deficiency conditions, which is consistent with previous study (Yang et al., 2020). It was reported that SPX domain proteins are essential for maintaining phosphorus homeostasis in plants (Secco et al., 2012; Wang et al., 2021). Two DEGs encoding SPX domain-containing protein 1 and two DEGs encoding SPX domain-containing membrane protein were down regulated under Ca^2+^ deficiency conditions (Supplementary Table 2). Obviously, Ca^2+^ deficiency not only affects intracellular Ca^2+^ homeostasis but also other ions transport and homeostasis such as Zn^2+^, Na^+^, K^+^, Mg^2+^, etc., and hinders the absorption of important peanut development needed nutrients. But the underlying mechanism for the complicated regulatory relationships among these ions is not clear. Our results poses an interesting research direction for further study.

### Some Key Transcription Regulators Responsible for Embryo Abortion

Several putative important regulatory genes were obtained that might play critical roles in peanut embryo abortion under conditions of calcium deficiency. Two APIFAs showed a sharp increase in calcium deficiency-treated embryos (Figures 4B, 6). These results implied that APIFA played a pivotal role in peanut embryo abortion under calcium deficiency. Apoptosis-inducing factor (AIF) is a phylogenetically conserved mitochondrial intermembrane flavoprotein that induces apoptosis *via* a caspase-independent pathway (Lorenzo et al., 1999). AIF is involved in the induction of nuclear chromatin condensation as well as large-scale DNA fragmentation (approximately 50 kb), and it is essential for programmed cell death during the cavitation of embryoid bodies (Lü et al., 2003). The up-regulation of APIFA indicated that apoptosis occurred in the early stages of peanut development under calcium-deficient conditions, an essential cause of the embryo abortion.

Several transcription factors (*TCP4*, *ANT*, *WRI1*, and *FUS3)* are related to embryogenesis or ovule development, and they were down-regulated in the calcium deficiency-treated embryos. *TCP4* is essential for post-zygotic development (Pagnussat et al., 2005). *ANT* plays a critical role in regulating ovule and female gametophyte development (Klucher et al., 1996). *FUS3* is an essential regulator of embryogenesis (Tsuchiya et al., 2004). *WRI1* is involved in the regulation of seed storage substance metabolism in Arabidopsis, especially seed oil accumulation (Cernac and Benning, 2004). The down-regulation of these transcription factors yields embryo development barriers under calcium deficiency.

## Data Availability Statement

The original contributions presented in the study are publicly available. This data can be found here: National Center for Biotechnology Information (NCBI) BioProject database under accession number PRJNA470988.

## Author Contributions

WZ and HC conceived the project, designed the research and conducted the experiments. HC performed most of the experiments, analyzed the data, and drafted the manuscript. QY carried out the data analysis and participated in drafting the manuscript. HF assisted in data collection and analysis. KC and SZ performed qRT-PCR analysis. CZ, TC, and LW participated in data collection and analysis. HD, MG, HL, and XY helped to prepare and take the samples. WZ and RV revised the manuscript. All authors have read, edited, and approved the current version of the manuscript.

## Conflict of Interest

The authors declare that the research was conducted in the absence of any commercial or financial relationships that could be construed as a potential conflict of interest.

## Publisher’s Note

All claims expressed in this article are solely those of the authors and do not necessarily represent those of their affiliated organizations, or those of the publisher, the editors and the reviewers. Any product that may be evaluated in this article, or claim that may be made by its manufacturer, is not guaranteed or endorsed by the publisher.

## Footnotes

1
http://peanutgr.fafu.edu.cn/

## Supplementary Material

Supplementary Figure 1 The percentage of mapped reads distribution in different sites (intro, intergenic and exon) of gene in peanut embryos under calcium deficiency and sufficiency conditions. The X axis represented three repetitions of peanut embryos under calcium deficiency and sufficiency conditions at 15, 20, and 30 DAP, respectively. The Y axis is represented the percentage of mapped reads distributed in different sites of gene. Blue column: exon; Orange: intergenic; Gray: intron. Three biological replicates were carried out for each sample.
