# Supplementary figures and images for: Identification of Key Gene Networks and Deciphering Transcriptional Regulators Associated With Peanut Embryo Abortion Mediated by Calcium Deficiency
Source: Front Plant Sci. 2022 Mar 21;13:814015. doi: 10.3389/fpls.2022.814015 (PMC8978587; doi:10.3389/fpls.2022.814015)

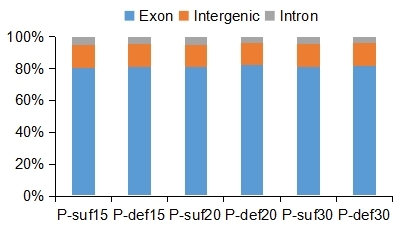

Supplement: Supplementary Figure 1 — The percentage of mapped reads distribution in different sites (intro, intergenic and exon) of gene in peanut embryos under calcium deficiency and sufficiency conditions. The X axis represented three repetitions of peanut embryos under calcium deficiency and sufficiency conditions at 15, 20, and 30 DAP, respectively. The Y axis is represented the percentage of mapped reads distributed in different sites of gene. Blue column: exon; Orange: intergenic; Gray: intron. Three biological replicates were carried out for each sample. [file Image_1.JPEG]

(A)

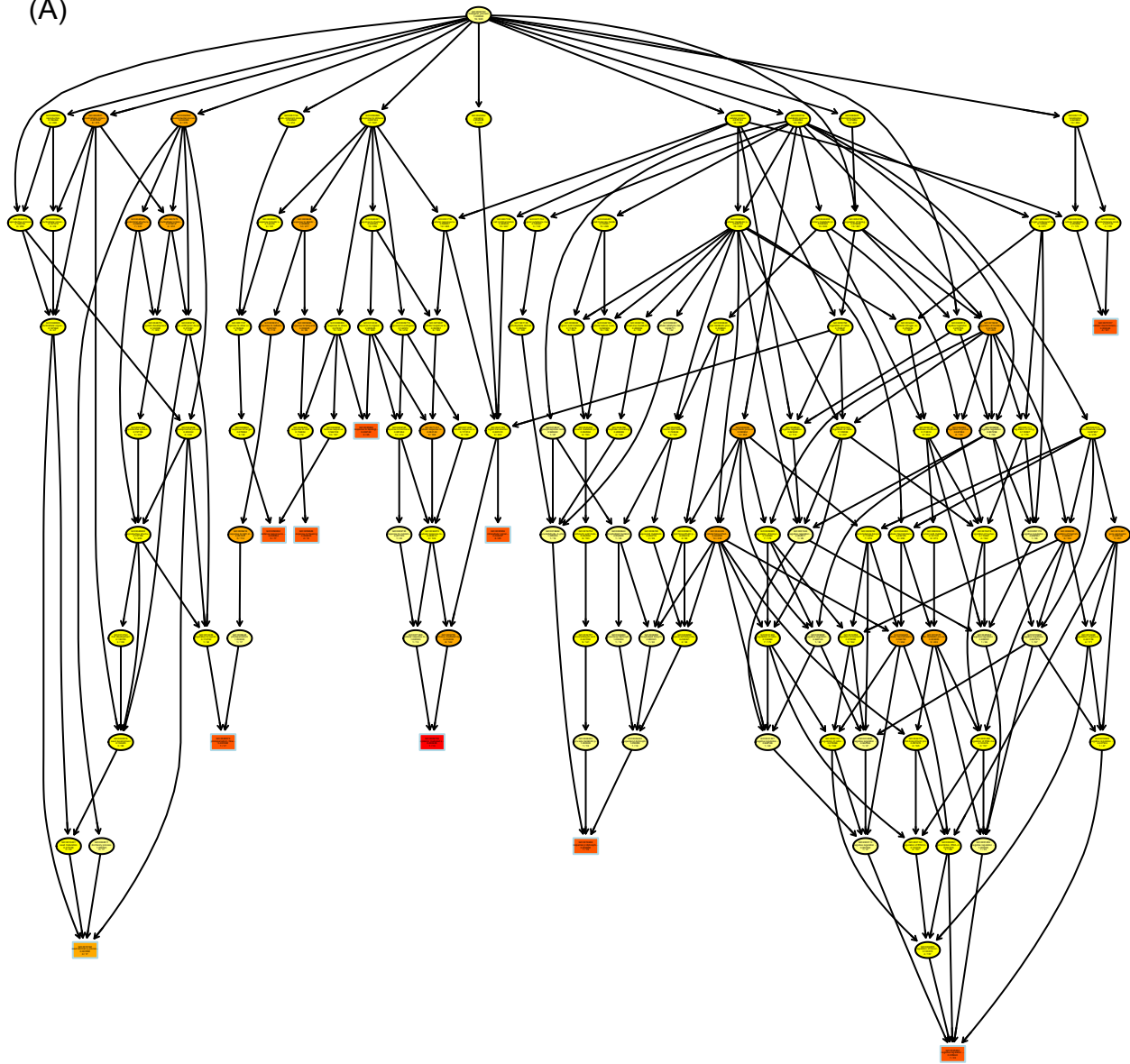

(B)

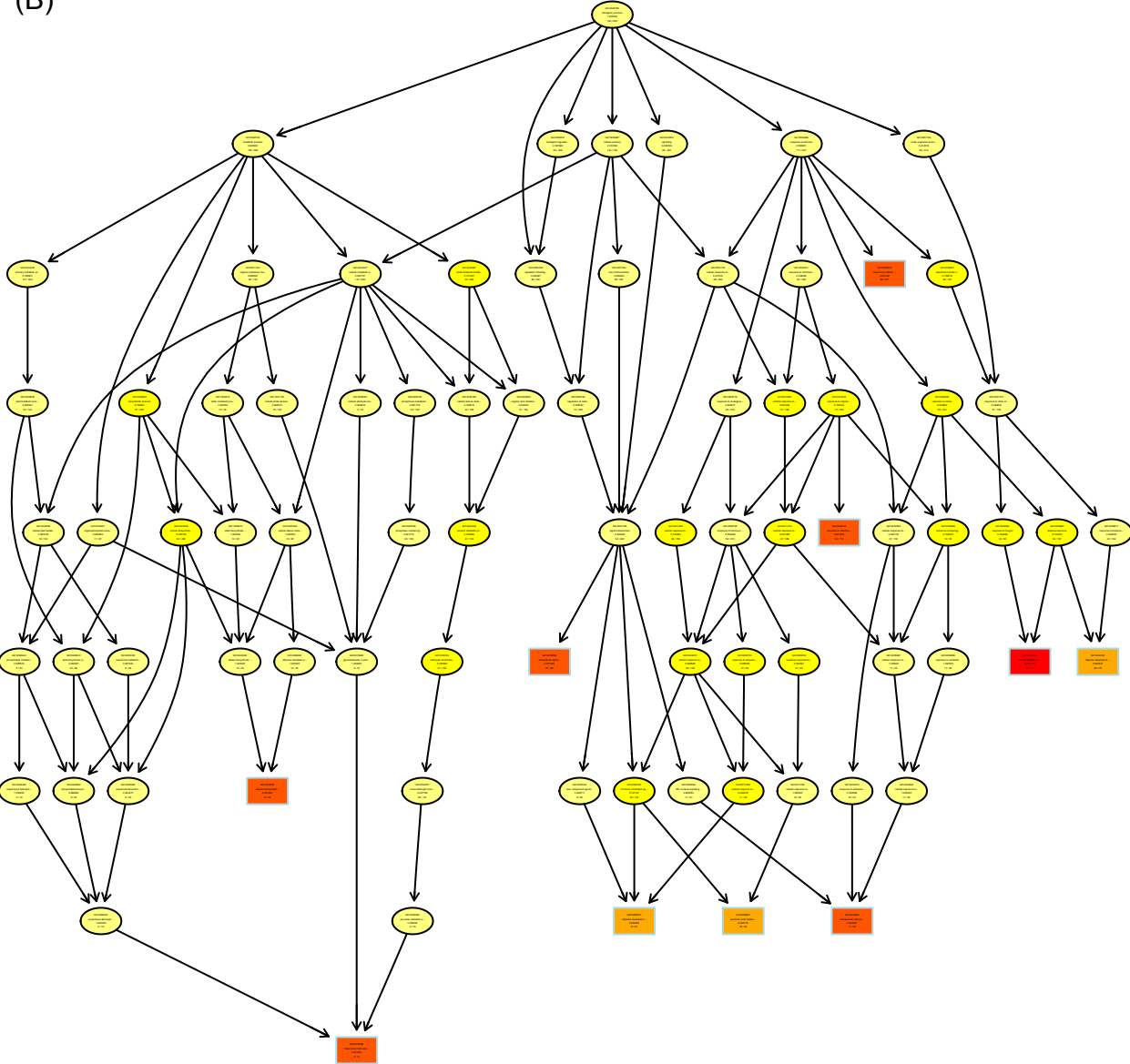

(C)

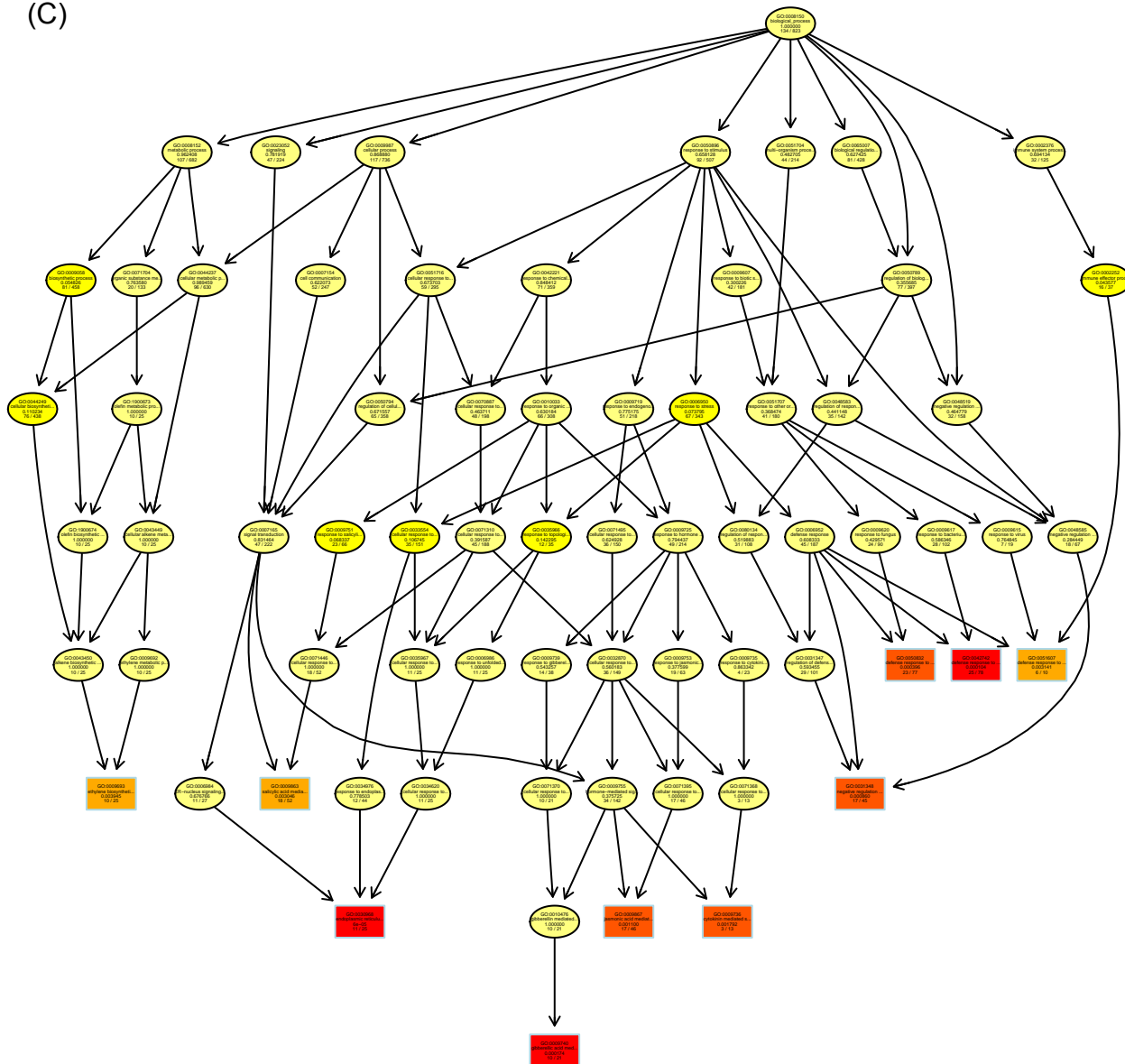

**Supplementary Figure 2 GO annotation-Biological process. (A)15DAP, (B)20DAP, (C)30DAP**

Supplement: Supplementary file 2 [file Image_2.PDF]

(A)

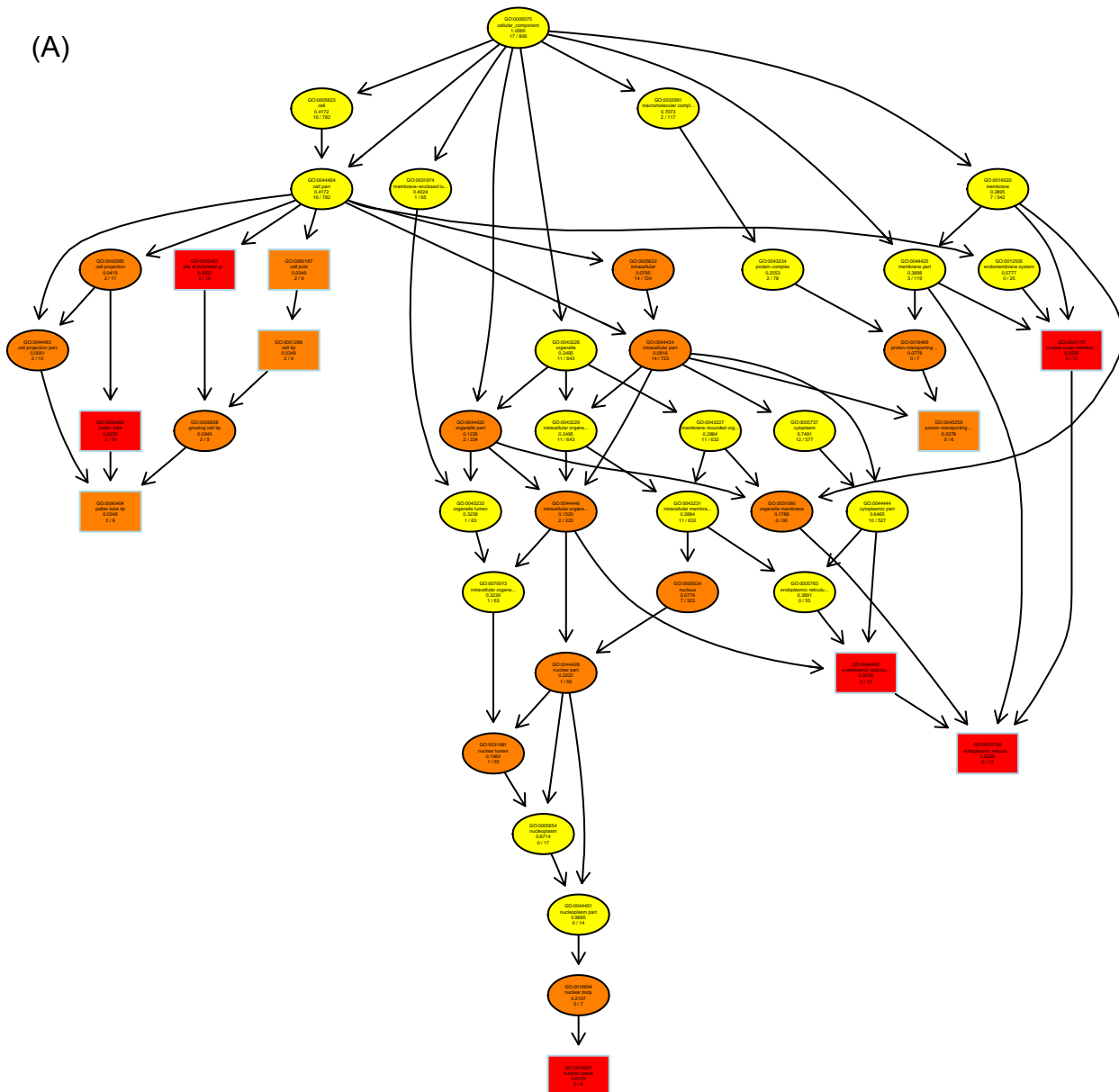

(B)

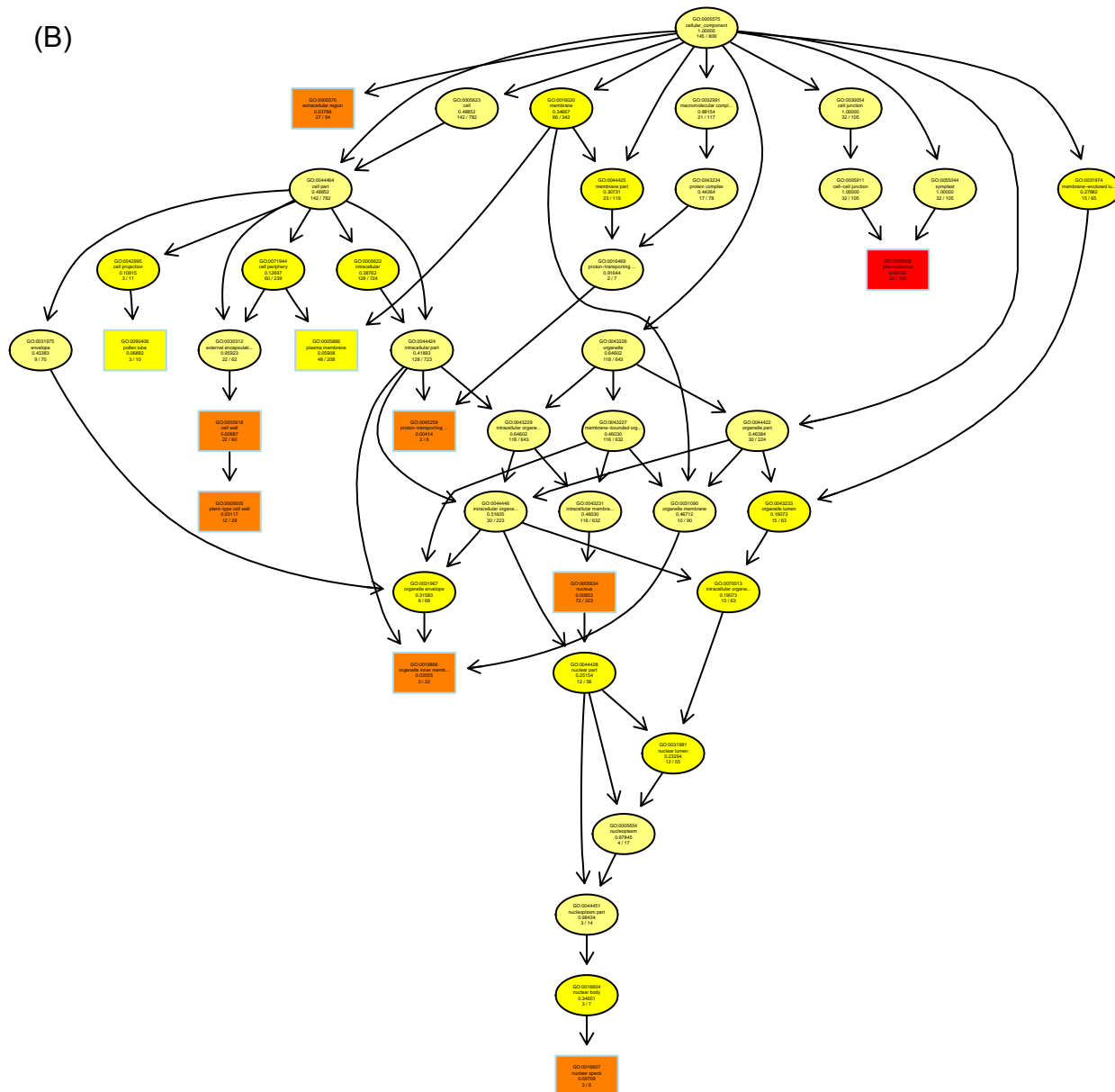

Supplement: Supplementary file 3 [file Image_3.PDF]

(A)

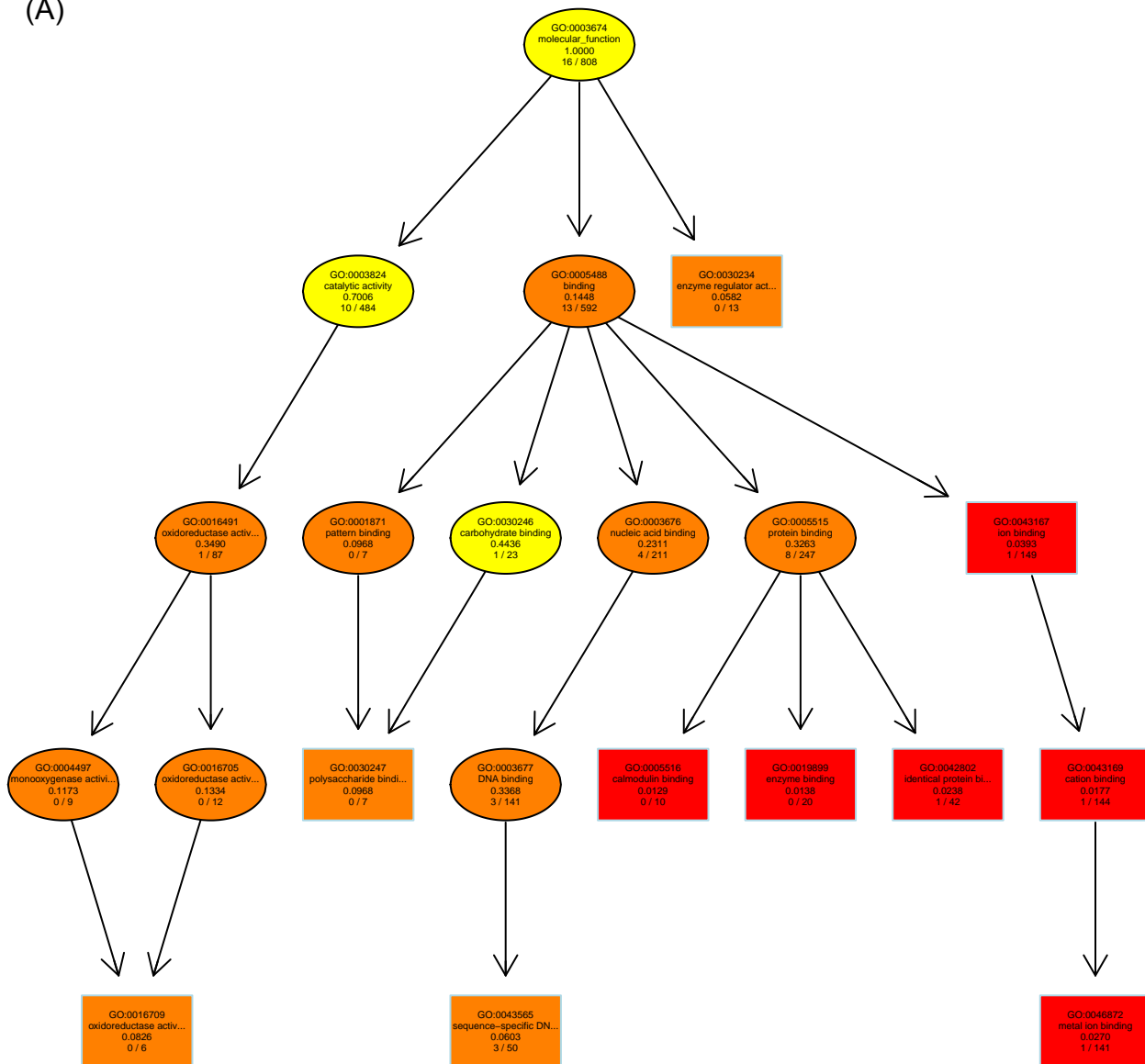

(B)

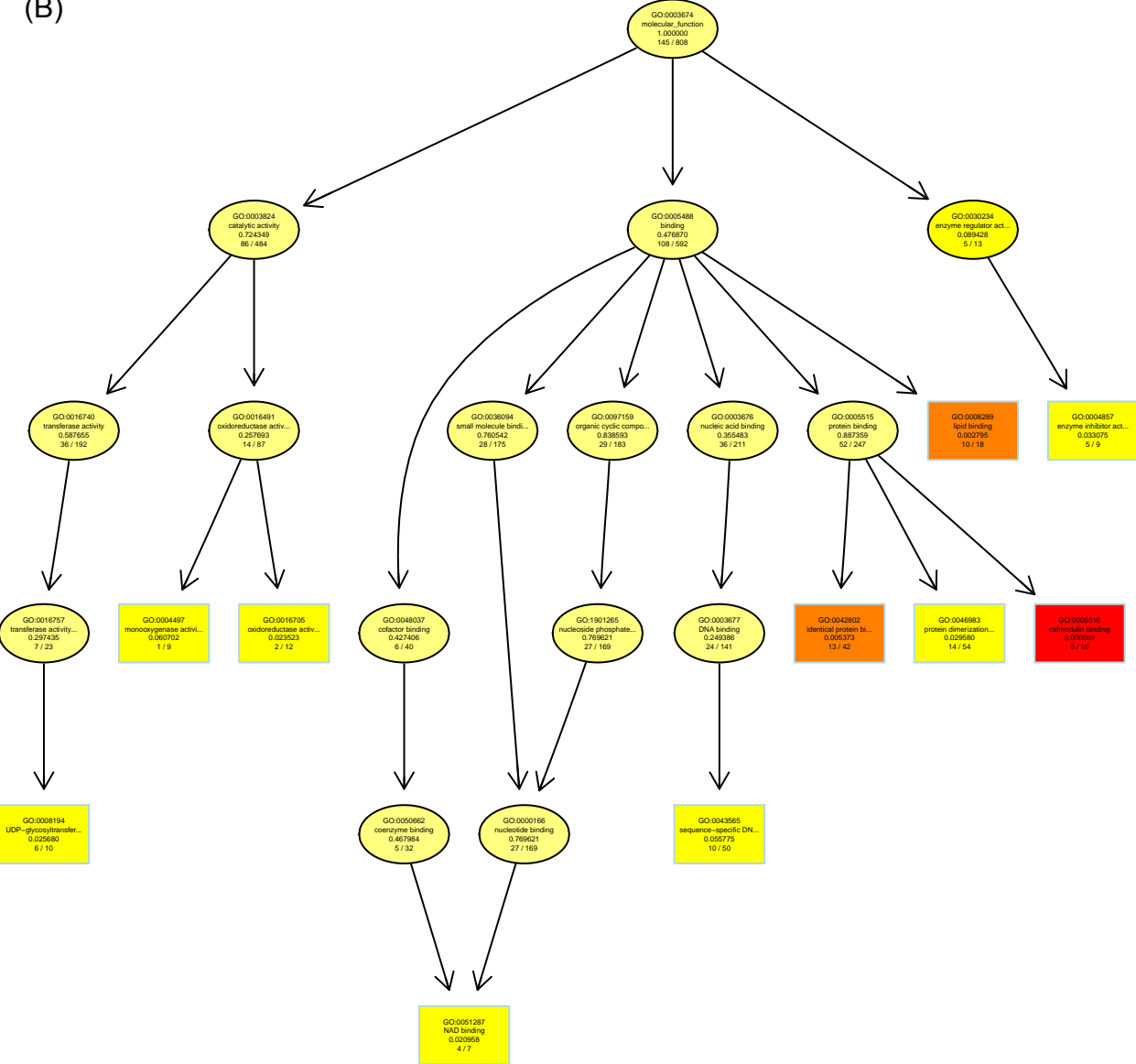

(C)

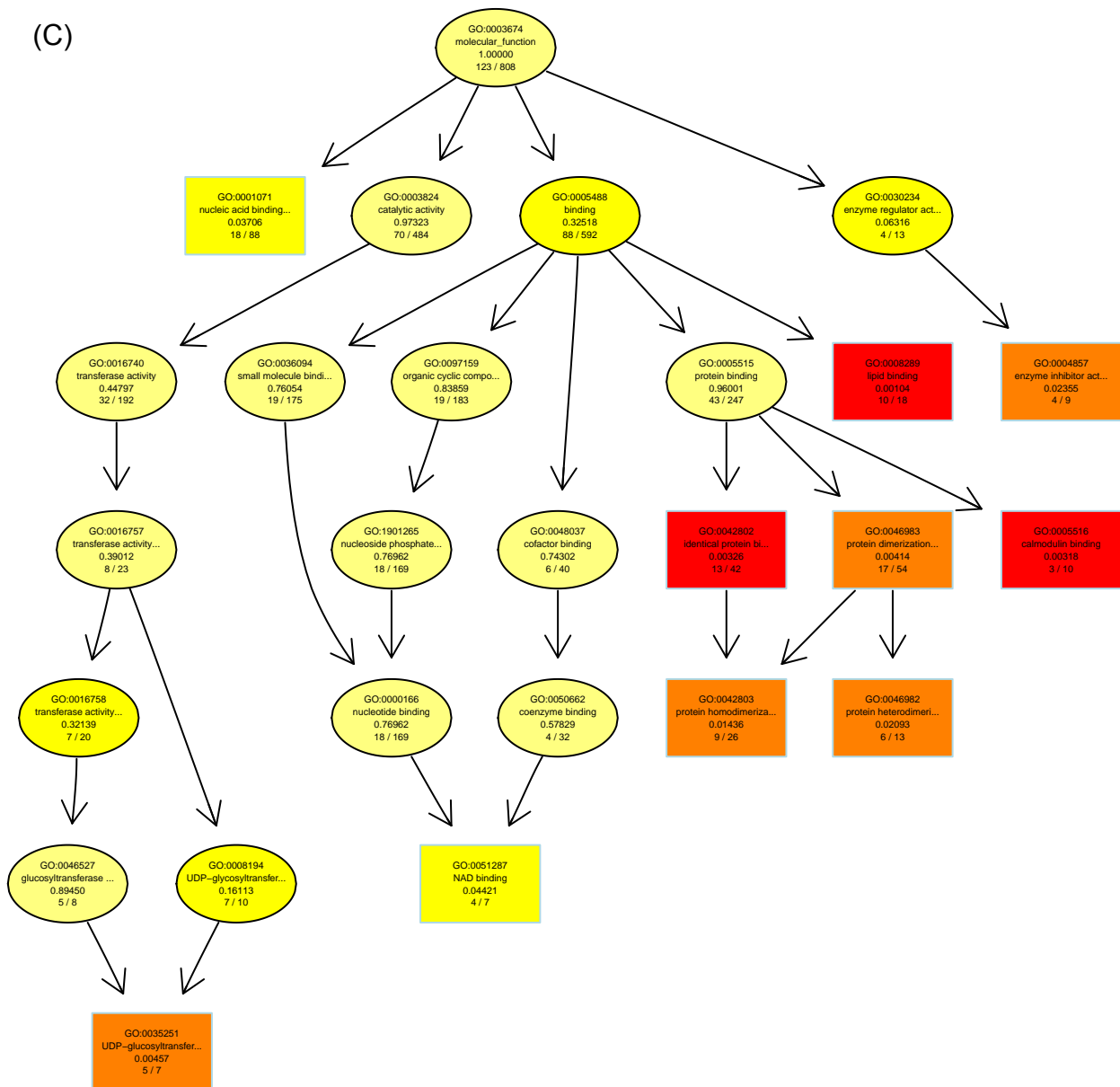

Supplementary Figure 4 GO annotation-Molecular function. (A)15DAP, (B)20DAP, (C)30DAP

Supplement: Supplementary file 4 [file Image_4.PDF]
