# Supplementary material for: Identification of Key Gene Networks and Deciphering Transcriptional Regulators Associated With Peanut Embryo Abortion Mediated by Calcium Deficiency
Source: Front Plant Sci. 2022 Mar 21;13:814015. doi: 10.3389/fpls.2022.814015 (PMC8978587; doi:10.3389/fpls.2022.814015)
Supplement: Supplementary file 5 [file Table_1.DOCX]

**Supplementary Table 1 Statistics of reads generated by RNA-seq analysis of peanut embryos under calcium deficiency and sufficiency conditions**

| **Samples** | **ReadSum** | **GC(%)** | **Q20(%)** | **Q30(%)** | **Total Reads** | **Mapped Reads** | **Uniq Mapped Reads** | **Multiple Map Reads** |
| --- | --- | --- | --- | --- | --- | --- | --- | --- |
| P-suf15 | 21212034 | 47.00 | 95.06 | 91.10 | 42424069 | 36276664 (85.70%) | 29304941 (71.14%) | 6971486 (14.56%) |
| P-def15 | 20012545 | 45.75 | 95.03 | 91.05 | 40025089 | 35610855 (89.11%) | 28008676 (77.69%) | 5842272 (11.42%) |
| P-suf20 | 22004325 | 47.29 | 94.85 | 90.76 | 44008651 | 37627482 (87.50%) | 30802175 (71.91%) | 6825307 (18.71%) |
| P-def20 | 22494487 | 45.62 | 95.30 | 91.06 | 44988973 | 38051341 (90.82%) | 32007208 (79.35%) | 6044133 (11.47%) |
| P-suf30 | 23951534 | 47.47 | 94.18 | 89.63 | 47903068 | 40422743 (90.02%) | 34004097 (70.44%) | 6418646 (19.59%) |
| P-def30 | 23655921 | 45.79 | 94.24 | 89.77 | 47311843 | 39338498 (91.49%) | 33774242 (79.39%) | 5564256 (12.09%) |
