# Supplementary material for: Identification of Key Gene Networks and Deciphering Transcriptional Regulators Associated With Peanut Embryo Abortion Mediated by Calcium Deficiency
Source: Front Plant Sci. 2022 Mar 21;13:814015. doi: 10.3389/fpls.2022.814015 (PMC8978587; doi:10.3389/fpls.2022.814015)
Supplement: Supplementary file 7 [file Table_3.docx]

**Supplementary Table 3 Biological processes TopGO enrichment results of DEGs**

| **GO ID** | **Term** | **Annotated** | **15DAP** | | | **20DAP** | |  | **30DAP** | | |
| --- | --- | --- | --- | --- | --- | --- | --- | --- | --- | --- | --- |
|  |  |  | **Significant** | **Expected** | **KS** | **Significant** | **Expected** | **KS** | **Significant** | **Expected** | **KS** |
| GO:0009058 | biosynthetic process | 458 | 14 | 10.57 | 0.0309 |  |  |  |  |  |  |
| GO:0044249 | cellular biosynthetic process | 438 | 14 | 10.11 | 0.04423 |  |  |  |  |  |  |
| GO:0009755 | hormone-mediated signaling pathway | 142 | 8 | 3.28 | 0.03401 |  |  |  |  |  |  |
| GO:0010035 | response to inorganic substance | 122 | 6 | 2.82 | 0.02298 |  |  |  |  |  |  |
| GO:0009653 | anatomical structure morphogenesis | 216 | 5 | 4.99 | 0.01275 |  |  |  |  |  |  |
| GO:0009753 | response to jasmonic acid | 63 | 5 | 1.45 | 0.01265 |  |  |  |  |  |  |
| GO:0006091 | generation of precursor metabolites and energy | 59 | 4 | 1.36 | 0.02807 |  |  |  |  |  |  |
| GO:0009657 | plastid organization | 62 | 4 | 1.43 | 0.02826 |  |  |  |  |  |  |
| GO:0010043 | response to zinc ion | 17 | 4 | 0.39 | 0.03187 |  |  |  |  |  |  |
| GO:0006073 | cellular glucan metabolic process | 35 | 3 | 0.81 | 0.0368 |  |  |  |  |  |  |
| GO:0035967 | cellular response to topologically incorrect protein | 25 | 3 | 0.58 | 0.01302 |  |  |  |  |  |  |
| GO:0034620 | cellular response to unfolded protein | 25 | 3 | 0.58 | 0.01302 |  |  |  |  |  |  |
| GO:0009658 | chloroplast organization | 31 | 3 | 0.72 | 0.019 |  |  |  |  |  |  |
| GO:0006984 | ER-nucleus signaling pathway | 27 | 3 | 0.62 | 0.03436 |  |  |  |  |  |  |
| GO:0009250 | glucan biosynthetic process | 27 | 3 | 0.62 | 0.04484 |  |  |  |  |  |  |
| GO:0050793 | regulation of developmental process | 131 | 3 | 3.02 | 0.03502 |  |  |  |  |  |  |
| GO:0035966 | response to topologically incorrect protein | 35 | 3 | 0.81 | 0.00212 |  |  |  |  |  |  |
| GO:0006986 | response to unfolded protein | 25 | 3 | 0.58 | 0.01302 |  |  |  |  |  |  |
| GO:1901137 | carbohydrate derivative biosynthetic process | 51 | 2 | 1.18 | 0.04521 |  |  |  |  |  |  |
| GO:0071470 | cellular response to osmotic stress | 10 | 2 | 0.23 | 0.0206 |  |  |  |  |  |  |
| GO:0071472 | cellular response to salt stress | 10 | 2 | 0.23 | 0.0206 |  |  |  |  |  |  |
| GO:0009639 | response to red or far red light | 42 | 2 | 0.97 | 0.01788 |  |  |  |  |  |  |
| GO:0019252 | starch biosynthetic process | 20 | 2 | 0.46 | 0.03667 |  |  |  |  |  |  |
| GO:0005982 | starch metabolic process | 24 | 2 | 0.55 | 0.03631 |  |  |  |  |  |  |
| GO:0046777 | protein autophosphorylation | 29 | 2 | 0.67 | 0.01541 |  |  |  |  |  |  |
| GO:0043450 | alkene biosynthetic process | 25 | 1 | 0.58 | 0.04787 |  |  |  |  |  |  |
| GO:0043449 | cellular alkene metabolic process | 25 | 1 | 0.58 | 0.04787 |  |  |  |  |  |  |
| GO:0071489 | cellular response to red or far red light | 12 | 1 | 0.28 | 0.0454 |  |  |  |  |  |  |
| GO:0009692 | ethylene metabolic process | 25 | 1 | 0.58 | 0.04787 |  |  |  |  |  |  |
| GO:0006887 | exocytosis | 7 | 1 | 0.16 | 0.01484 |  |  |  |  |  |  |
| GO:0016138 | glycoside biosynthetic process | 30 | 1 | 0.69 | 0.04439 |  |  |  |  |  |  |
| GO:0016137 | glycoside metabolic process | 33 | 1 | 0.76 | 0.04612 |  |  |  |  |  |  |
| GO:1900674 | olefin biosynthetic process | 25 | 1 | 0.58 | 0.04787 |  |  |  |  |  |  |
| GO:1900673 | olefin metabolic process | 25 | 1 | 0.58 | 0.04787 |  |  |  |  |  |  |
| GO:0009668 | plastid membrane organization | 26 | 1 | 0.6 | 0.03472 |  |  |  |  |  |  |
| GO:0010017 | red or far-red light signaling pathway | 12 | 1 | 0.28 | 0.0454 |  |  |  |  |  |  |
| GO:0032940 | secretion by cell | 10 | 1 | 0.23 | 0.0087 |  |  |  |  |  |  |
| GO:0010027 | thylakoid membrane organization | 26 | 1 | 0.6 | 0.03472 |  |  |  |  |  |  |
| GO:0002252 | immune effector process | 37 | 5 | 0.85 | 0.02907 |  |  |  | 16 | 6.02 | 0.04358 |
| GO:0045730 | respiratory burst | 29 | 4 | 0.67 | 0.04783 |  |  |  | 11 | 4.72 | 0.04088 |
| GO:0071369 | cellular response to ethylene stimulus | 28 | 3 | 0.65 | 0.00987 |  |  |  | 8 | 4.56 | 0.00739 |
| GO:0002253 | activation of immune response | 10 | 2 | 0.23 | 0.02922 |  |  |  | 5 | 1.63 | 0.01901 |
| GO:0009736 | cytokinin-activated signaling pathway | 13 | 1 | 0.3 | 0.00025 |  |  |  | 3 | 2.12 | 0.00179 |
| GO:0019760 | glucosinolate metabolic process | 25 | 1 | 0.58 | 0.01549 |  |  |  | 5 | 4.07 | 0.03394 |
| GO:0019758 | glycosinolate biosynthetic process | 22 | 1 | 0.51 | 0.01308 |  |  |  | 5 | 3.58 | 0.0209 |
| GO:0019757 | glycosinolate metabolic process | 25 | 1 | 0.58 | 0.01549 |  |  |  | 5 | 4.07 | 0.03394 |
| GO:0034660 | ncRNA metabolic process | 25 | 1 | 0.58 | 0.03029 |  |  |  | 5 | 4.07 | 0.02122 |
| GO:0048573 | photoperiodism, flowering | 17 | 1 | 0.39 | 0.00741 |  |  |  | 5 | 2.77 | 0.04876 |
| GO:0009628 | response to abiotic stimulus | 277 | 12 | 6.39 | 0.00888 | 69 | 50.49 | 0.00134 |  |  |  |
| GO:0019222 | regulation of metabolic process | 233 | 10 | 5.38 | 0.04104 | 56 | 42.47 | 0.00408 |  |  |  |
| GO:0009743 | response to carbohydrate | 110 | 10 | 2.54 | 0.0306 | 32 | 20.05 | 0.00135 | 29 | 17.91 | 0.00658 |
| GO:0032502 | developmental process | 379 | 9 | 8.75 | 0.0433 | 82 | 69.08 | 0.01685 | 70 | 61.71 | 0.02948 |
| GO:0080090 | regulation of primary metabolic process | 193 | 8 | 4.46 | 0.04788 | 39 | 35.18 | 0.02631 |  |  |  |
| GO:0044283 | small molecule biosynthetic process | 135 | 7 | 3.12 | 0.01976 | 35 | 24.61 | 0.01265 |  |  |  |
| GO:0046394 | carboxylic acid biosynthetic process | 122 | 6 | 2.82 | 0.03556 | 32 | 22.24 | 0.03162 |  |  |  |
| GO:0042742 | defense response to bacterium | 78 | 6 | 1.8 | 0.0099 | 28 | 14.22 | 0.00244 | 25 | 12.7 | 0.0001 |
| GO:0035556 | intracellular signal transduction | 85 | 6 | 1.96 | 0.00426 | 27 | 15.49 | 0.00149 | 21 | 13.84 | 0.00919 |
| GO:0016053 | organic acid biosynthetic process | 122 | 6 | 2.82 | 0.03556 | 32 | 22.24 | 0.03162 |  |  |  |
| GO:0060255 | regulation of macromolecule metabolic process | 184 | 6 | 4.25 | 0.04698 | 35 | 33.54 | 0.01264 | 38 | 29.96 | 0.04049 |
| GO:0009266 | response to temperature stimulus | 99 | 6 | 2.29 | 0.03683 | 27 | 18.04 | 0.03436 | 20 | 16.12 | 0.04169 |
| GO:0050832 | defense response to fungus | 77 | 5 | 1.78 | 0.00486 | 27 | 14.03 | 0.000062 | 23 | 12.54 | 0.0004 |
| GO:0016070 | RNA metabolic process | 233 | 5 | 5.38 | 0.03389 | 34 | 42.47 | 0.03235 | 37 | 37.94 | 0.04841 |
| GO:0070727 | cellular macromolecule localization | 121 | 4 | 2.79 | 0.00635 | 28 | 22.05 | 0.0138 |  |  |  |
| GO:0034613 | cellular protein localization | 110 | 4 | 2.54 | 0.01958 | 23 | 20.05 | 0.04106 |  |  |  |
| GO:0045184 | establishment of protein localization | 111 | 4 | 2.56 | 0.02542 | 22 | 20.23 | 0.03232 |  |  |  |
| GO:0006886 | intracellular protein transport | 104 | 4 | 2.4 | 0.02208 | 21 | 18.96 | 0.03548 |  |  |  |
| GO:0006811 | ion transport | 84 | 4 | 1.94 | 0.04884 | 19 | 15.31 | 0.01459 | 15 | 13.68 | 0.04152 |
| GO:0006605 | protein targeting | 99 | 4 | 2.29 | 0.01806 | 21 | 18.04 | 0.02322 |  |  |  |
| GO:0015031 | protein transport | 111 | 4 | 2.56 | 0.02542 | 22 | 20.23 | 0.03232 |  |  |  |
| GO:0002679 | respiratory burst involved in defense response | 29 | 4 | 0.67 | 0.04783 | 13 | 5.29 | 0.00342 | 11 | 4.72 | 0.04088 |
| GO:0009415 | response to water | 70 | 4 | 1.62 | 0.0375 | 20 | 12.76 | 0.01069 |  |  |  |
| GO:0009414 | response to water deprivation | 70 | 4 | 1.62 | 0.0375 | 20 | 12.76 | 0.01069 |  |  |  |
| GO:0030968 | endoplasmic reticulum unfolded protein response | 25 | 3 | 0.58 | 0.01302 | 11 | 4.56 | 0.00047 | 11 | 4.07 | 6.00E-05 |
| GO:0006007 | glucose catabolic process | 58 | 3 | 1.34 | 0.00813 | 11 | 10.57 | 0.02319 |  |  |  |
| GO:0009637 | response to blue light | 12 | 3 | 0.28 | 0.02207 | 4 | 2.19 | 0.03995 | 4 | 1.95 | 0.0307 |
| GO:0002218 | activation of innate immune response | 10 | 2 | 0.23 | 0.02922 | 6 | 1.82 | 0.00364 | 5 | 1.63 | 0.01901 |
| GO:0009873 | ethylene-activated signaling pathway | 22 | 2 | 0.51 | 0.03334 | 9 | 4.01 | 0.00284 | 7 | 3.58 | 0.01997 |
| GO:0015979 | photosynthesis | 36 | 2 | 0.83 | 0.01076 | 9 | 6.56 | 0.01061 | 8 | 5.86 | 0.04259 |
| GO:0050826 | response to freezing | 12 | 2 | 0.28 | 0.00693 | 3 | 2.19 | 0.00866 | 4 | 1.95 | 0.03539 |
| GO:0000041 | transition metal ion transport | 26 | 2 | 0.6 | 0.02326 | 5 | 4.74 | 0.0489 |  |  |  |
| GO:0007568 | aging | 37 | 1 | 0.85 | 0.02203 | 11 | 6.74 | 0.04811 | 11 | 6.02 | 0.00428 |
| GO:0016101 | diterpenoid metabolic process | 9 | 1 | 0.21 | 0.03846 | 2 | 1.64 | 0.04982 |  |  |  |
| GO:0009693 | ethylene biosynthetic process | 25 | 1 | 0.58 | 0.04787 | 12 | 4.56 | 0.00159 | 10 | 4.07 | 0.00395 |
| GO:0019761 | glucosinolate biosynthetic process | 22 | 1 | 0.51 | 0.01308 | 6 | 4.01 | 0.00824 | 5 | 3.58 | 0.0209 |
| GO:0019288 | isopentenyl diphosphate biosynthetic process, methylerythritol 4-phosphate pathway | 19 | 1 | 0.44 | 0.00299 | 4 | 3.46 | 0.00184 | 2 | 3.09 | 0.02288 |
| GO:0045892 | negative regulation of transcription, DNA-templated | 33 | 1 | 0.76 | 0.00562 | 6 | 6.01 | 0.01362 | 8 | 5.37 | 0.00514 |
| GO:0048580 | regulation of post-embryonic development | 78 | 1 | 1.8 | 0.03065 | 20 | 14.22 | 0.03546 | 17 | 12.7 | 0.04828 |
| GO:0002831 | regulation of response to biotic stimulus | 7 | 1 | 0.16 | 0.04276 | 2 | 1.28 | 0.03141 |  |  |  |
| GO:0009845 | seed germination | 42 | 1 | 0.97 | 0.01755 | 12 | 7.65 | 0.01647 |  |  |  |
| GO:0090351 | seedling development | 44 | 1 | 1.02 | 0.0392 | 12 | 8.02 | 0.04296 |  |  |  |
| GO:0016144 | S-glycoside biosynthetic process | 22 | 1 | 0.51 | 0.01308 |  |  |  | 5 | 3.58 | 0.0209 |
| GO:0016143 | S-glycoside metabolic process | 25 | 1 | 0.58 | 0.01549 |  |  |  | 5 | 4.07 | 0.03394 |
| GO:0010182 | sugar mediated signaling pathway | 9 | 1 | 0.21 | 0.00924 | 1 | 1.64 | 0.04433 | 2 | 1.47 | 0.03681 |
| GO:0006950 | response to stress | 343 |  |  |  | 85 | 62.52 | 0.04285 |  |  |  |
| GO:0007275 | multicellular organismal development | 372 |  |  |  | 81 | 67.8 | 0.02996 | 69 | 60.57 | 0.03216 |
| GO:0032501 | multicellular organismal process | 375 |  |  |  | 81 | 68.35 | 0.02133 |  |  |  |
| GO:0051179 | localization | 284 |  |  |  | 62 | 51.76 | 0.02736 |  |  |  |
| GO:0051234 | establishment of localization | 263 |  |  |  | 55 | 47.93 | 0.04862 |  |  |  |
| GO:0009791 | post-embryonic development | 249 |  |  |  | 54 | 45.38 | 0.01894 | 46 | 40.54 | 0.0236 |
| GO:0031323 | regulation of cellular metabolic process | 207 |  |  |  | 47 | 37.73 | 0.01834 |  |  |  |
| GO:0010467 | gene expression | 251 |  |  |  | 40 | 45.75 | 0.03196 |  |  |  |
| GO:0009889 | regulation of biosynthetic process | 179 |  |  |  | 37 | 32.62 | 0.0147 |  |  |  |
| GO:0031326 | regulation of cellular biosynthetic process | 179 |  |  |  | 37 | 32.62 | 0.0147 |  |  |  |
| GO:0006725 | cellular aromatic compound metabolic process | 118 |  |  |  | 36 | 21.51 | 0.02566 |  |  |  |
| GO:0033036 | macromolecule localization | 155 |  |  |  | 36 | 28.25 | 0.02835 |  |  |  |
| GO:2000112 | regulation of cellular macromolecule biosynthetic process | 162 |  |  |  | 32 | 29.53 | 0.01605 | 33 | 26.38 | 0.04208 |
| GO:0010556 | regulation of macromolecule biosynthetic process | 162 |  |  |  | 32 | 29.53 | 0.01605 | 33 | 26.38 | 0.04208 |
| GO:0009737 | response to abscisic acid | 112 |  |  |  | 32 | 20.41 | 0.00732 | 30 | 18.24 | 0.01541 |
| GO:0051171 | regulation of nitrogen compound metabolic process | 170 |  |  |  | 32 | 30.98 | 0.01951 |  |  |  |
| GO:0010468 | regulation of gene expression | 171 |  |  |  | 31 | 31.17 | 0.02584 |  |  |  |
| GO:0031347 | regulation of defense response | 101 |  |  |  | 31 | 18.41 | 0.033 |  |  |  |
| GO:0051252 | regulation of RNA metabolic process | 157 |  |  |  | 29 | 28.61 | 0.02997 |  |  |  |
| GO:2001141 | regulation of RNA biosynthetic process | 157 |  |  |  | 29 | 28.61 | 0.02997 |  |  |  |
| GO:0006355 | regulation of transcription, DNA-templated | 157 |  |  |  | 29 | 28.61 | 0.02997 |  |  |  |
| GO:0019438 | aromatic compound biosynthetic process | 91 |  |  |  | 29 | 16.59 | 0.03779 |  |  |  |
| GO:0019219 | regulation of nucleobase-containing compound metabolic process | 162 |  |  |  | 29 | 29.53 | 0.03808 |  |  |  |
| GO:0006351 | transcription, DNA-templated | 165 |  |  |  | 29 | 30.07 | 0.04091 |  |  |  |
| GO:0032774 | RNA biosynthetic process | 165 |  |  |  | 29 | 30.07 | 0.04091 |  |  |  |
| GO:0065008 | regulation of biological quality | 117 |  |  |  | 29 | 21.32 | 0.04173 |  |  |  |
| GO:0009605 | response to external stimulus | 100 |  |  |  | 27 | 18.23 | 0.01603 |  |  |  |
| GO:0009416 | response to light stimulus | 114 |  |  |  | 26 | 20.78 | 0.02658 | 29 | 18.56 | 0.00403 |
| GO:0009314 | response to radiation | 114 |  |  |  | 26 | 20.78 | 0.02658 |  |  |  |
| GO:0010941 | regulation of cell death | 85 |  |  |  | 25 | 15.49 | 0.00528 | 23 | 13.84 | 0.00441 |
| GO:0012501 | programmed cell death | 88 |  |  |  | 23 | 16.04 | 0.02604 |  |  |  |
| GO:0009814 | defense response, incompatible interaction | 70 |  |  |  | 21 | 12.76 | 0.00998 | 19 | 11.4 | 0.01437 |
| GO:0043067 | regulation of programmed cell death | 79 |  |  |  | 21 | 14.4 | 0.02294 | 18 | 12.86 | 0.03903 |
| GO:0009751 | response to salicylic acid | 66 |  |  |  | 21 | 12.03 | 0.00369 |  |  |  |
| GO:0010200 | response to chitin | 69 |  |  |  | 21 | 12.58 | 0.02948 |  |  |  |
| GO:0048585 | negative regulation of response to stimulus | 67 |  |  |  | 19 | 12.21 | 0.00805 |  |  |  |
| GO:0071215 | cellular response to abscisic acid stimulus | 54 |  |  |  | 18 | 9.84 | 0.03552 |  |  |  |
| GO:0048438 | floral whorl development | 72 |  |  |  | 17 | 13.12 | 0.04402 | 16 | 11.72 | 0.02099 |
| GO:0009738 | abscisic acid-activated signaling pathway | 47 |  |  |  | 17 | 8.57 | 0.01925 |  |  |  |
| GO:0009863 | salicylic acid mediated signaling pathway | 52 |  |  |  | 16 | 9.48 | 0.03285 | 18 | 8.47 | 0.00305 |
| GO:0031348 | negative regulation of defense response | 45 |  |  |  | 16 | 8.2 | 0.01056 | 17 | 7.33 | 0.00086 |
| GO:0009867 | jasmonic acid mediated signaling pathway | 46 |  |  |  | 16 | 8.38 | 0.00278 | 17 | 7.49 | 0.0011 |
| GO:0071446 | cellular response to salicylic acid stimulus | 52 |  |  |  | 16 | 9.48 | 0.03285 |  |  |  |
| GO:2000377 | regulation of reactive oxygen species metabolic process | 35 |  |  |  | 13 | 6.38 | 0.00791 | 13 | 5.7 | 0.0045 |
| GO:0031667 | response to nutrient levels | 44 |  |  |  | 13 | 8.02 | 0.02166 |  |  |  |
| GO:0006812 | cation transport | 56 |  |  |  | 13 | 10.21 | 0.0318 |  |  |  |
| GO:0042445 | hormone metabolic process | 39 |  |  |  | 12 | 7.11 | 0.01969 | 13 | 6.35 | 0.00452 |
| GO:0009862 | systemic acquired resistance, salicylic acid mediated signaling pathway | 37 |  |  |  | 12 | 6.74 | 0.02184 | 12 | 6.02 | 0.01692 |
| GO:0009697 | salicylic acid biosynthetic process | 31 |  |  |  | 12 | 5.65 | 0.00483 | 11 | 5.05 | 0.00912 |
| GO:0006865 | amino acid transport | 40 |  |  |  | 12 | 7.29 | 0.02873 |  |  |  |
| GO:0010310 | regulation of hydrogen peroxide metabolic process | 33 |  |  |  | 11 | 6.01 | 0.02974 | 11 | 5.37 | 0.01862 |
| GO:0042594 | response to starvation | 37 |  |  |  | 11 | 6.74 | 0.01387 |  |  |  |
| GO:0046365 | monosaccharide catabolic process | 58 |  |  |  | 11 | 10.57 | 0.02319 |  |  |  |
| GO:0019320 | hexose catabolic process | 58 |  |  |  | 11 | 10.57 | 0.02319 |  |  |  |
| GO:0005996 | monosaccharide metabolic process | 64 |  |  |  | 11 | 11.66 | 0.04161 |  |  |  |
| GO:0019318 | hexose metabolic process | 63 |  |  |  | 11 | 11.48 | 0.04574 |  |  |  |
| GO:0009267 | cellular response to starvation | 34 |  |  |  | 10 | 6.2 | 0.03324 |  |  |  |
| GO:0031669 | cellular response to nutrient levels | 34 |  |  |  | 10 | 6.2 | 0.03324 |  |  |  |
| GO:0009813 | flavonoid biosynthetic process | 25 |  |  |  | 10 | 4.56 | 0.03582 |  |  |  |
| GO:0009812 | flavonoid metabolic process | 26 |  |  |  | 10 | 4.74 | 0.04878 |  |  |  |
| GO:0032504 | multicellular organism reproduction | 49 |  |  |  | 9 | 8.93 | 0.01656 | 12 | 7.98 | 0.00987 |
| GO:0051606 | detection of stimulus | 28 |  |  |  | 9 | 5.1 | 0.02096 | 9 | 4.56 | 0.00868 |
| GO:0030001 | metal ion transport | 37 |  |  |  | 9 | 6.74 | 0.0334 |  |  |  |
| GO:0048609 | multicellular organismal reproductive process | 47 |  |  |  | 8 | 8.57 | 0.03361 | 11 | 7.65 | 0.02118 |
| GO:0009069 | serine family amino acid metabolic process | 23 |  |  |  | 8 | 4.19 | 0.01873 | 8 | 3.74 | 0.01079 |
| GO:0010629 | negative regulation of gene expression | 47 |  |  |  | 8 | 8.57 | 0.0133 |  |  |  |
| GO:0010228 | vegetative to reproductive phase transition of meristem | 38 |  |  |  | 7 | 6.93 | 0.04049 | 9 | 6.19 | 0.01102 |
| GO:0007166 | cell surface receptor signaling pathway | 17 |  |  |  | 7 | 3.1 | 0.03691 | 7 | 2.77 | 0.0261 |
| GO:0009612 | response to mechanical stimulus | 13 |  |  |  | 7 | 2.37 | 0.01402 |  |  |  |
| GO:0034754 | cellular hormone metabolic process | 19 |  |  |  | 7 | 3.46 | 0.02942 |  |  |  |
| GO:0009740 | gibberellic acid mediated signaling pathway | 21 |  |  |  | 6 | 3.83 | 0.02616 | 10 | 3.42 | 0.00017 |
| GO:0010431 | seed maturation | 30 |  |  |  | 6 | 5.47 | 0.04286 | 10 | 4.88 | 0.03022 |
| GO:0051607 | defense response to virus | 10 |  |  |  | 6 | 1.82 | 0.00466 | 6 | 1.63 | 0.00314 |
| GO:0009070 | serine family amino acid biosynthetic process | 18 |  |  |  | 6 | 3.28 | 0.02801 | 6 | 2.93 | 0.01769 |
| GO:0019344 | cysteine biosynthetic process | 18 |  |  |  | 6 | 3.28 | 0.02801 | 6 | 2.93 | 0.01769 |
| GO:0006534 | cysteine metabolic process | 19 |  |  |  | 6 | 3.46 | 0.04212 | 6 | 3.09 | 0.02696 |
| GO:0051253 | negative regulation of RNA metabolic process | 33 |  |  |  | 6 | 6.01 | 0.01362 |  |  |  |
| GO:0006869 | lipid transport | 12 |  |  |  | 6 | 2.19 | 0.02016 |  |  |  |
| GO:0071370 | cellular response to gibberellin stimulus | 21 |  |  |  | 6 | 3.83 | 0.02616 |  |  |  |
| GO:0010476 | gibberellin mediated signaling pathway | 21 |  |  |  | 6 | 3.83 | 0.02616 |  |  |  |
| GO:0019932 | second-messenger-mediated signaling | 17 |  |  |  | 6 | 3.1 | 0.03888 |  |  |  |
| GO:0006733 | oxidoreduction coenzyme metabolic process | 28 |  |  |  | 6 | 5.1 | 0.04778 |  |  |  |
| GO:0010286 | heat acclimation | 13 |  |  |  | 6 | 2.37 | 0.04787 |  |  |  |
| GO:0009850 | auxin metabolic process | 16 |  |  |  | 5 | 2.92 | 0.019 | 5 | 2.61 | 0.01394 |
| GO:0009682 | induced systemic resistance | 10 |  |  |  | 5 | 1.82 | 0.00364 | 5 | 1.63 | 0.01901 |
| GO:0042435 | indole-containing compound biosynthetic process | 16 |  |  |  | 5 | 2.92 | 0.01562 | 5 | 2.61 | 0.04426 |
| GO:0034220 | ion transmembrane transport | 19 |  |  |  | 5 | 3.46 | 0.03544 |  |  |  |
| GO:0022611 | dormancy process | 17 |  |  |  | 4 | 3.1 | 0.01477 | 7 | 2.77 | 0.01095 |
| GO:0010162 | seed dormancy process | 17 |  |  |  | 4 | 3.1 | 0.01477 | 7 | 2.77 | 0.01095 |
| GO:0035821 | modification of morphology or physiology of other organism | 8 |  |  |  | 4 | 1.46 | 0.04437 | 4 | 1.3 | 0.02854 |
| GO:0009683 | indoleacetic acid metabolic process | 10 |  |  |  | 4 | 1.82 | 0.01153 |  |  |  |
| GO:0009684 | indoleacetic acid metabolic process | 10 |  |  |  | 4 | 1.82 | 0.01153 |  |  |  |
| GO:0009749 | response to glucose | 11 |  |  |  | 4 | 2 | 0.01935 |  |  |  |
| GO:0001666 | response to hypoxia | 6 |  |  |  | 4 | 1.09 | 0.02259 |  |  |  |
| GO:0043255 | regulation of carbohydrate biosynthetic process | 7 |  |  |  | 3 | 1.28 | 0.04276 | 4 | 1.14 | 0.02625 |
| GO:0032412 | regulation of ion transmembrane transporter activity | 7 |  |  |  | 3 | 1.28 | 0.02913 | 3 | 1.14 | 0.02322 |
| GO:0010359 | regulation of anion channel activity | 7 |  |  |  | 3 | 1.28 | 0.02913 | 3 | 1.14 | 0.02322 |
| GO:0032409 | regulation of transporter activity | 7 |  |  |  | 3 | 1.28 | 0.02913 | 3 | 1.14 | 0.02322 |
| GO:0044070 | regulation of anion transport | 7 |  |  |  | 3 | 1.28 | 0.02913 | 3 | 1.14 | 0.02322 |
| GO:0022898 | regulation of transmembrane transporter activity | 7 |  |  |  | 3 | 1.28 | 0.02913 | 3 | 1.14 | 0.02322 |
| GO:0034762 | regulation of transmembrane transport | 7 |  |  |  | 3 | 1.28 | 0.02913 | 3 | 1.14 | 0.02322 |
| GO:0034765 | regulation of ion transmembrane transport | 7 |  |  |  | 3 | 1.28 | 0.02913 | 3 | 1.14 | 0.02322 |
| GO:0000398 | mRNA splicing, via spliceosome | 11 |  |  |  | 3 | 2 | 0.01522 | 2 | 1.79 | 0.01116 |
| GO:0000375 | RNA splicing, via transesterification reactions | 12 |  |  |  | 3 | 2.19 | 0.03399 | 2 | 1.95 | 0.02536 |
| GO:0000377 | RNA splicing, via transesterification reactions with bulged adenosine as nucleophile | 12 |  |  |  | 3 | 2.19 | 0.03399 | 2 | 1.95 | 0.02536 |
| GO:0015992 | proton transport | 11 |  |  |  | 3 | 2 | 0.02624 |  |  |  |
| GO:0006818 | hydrogen transport | 11 |  |  |  | 3 | 2 | 0.02624 |  |  |  |
| GO:0009686 | gibberellin biosynthetic process | 7 |  |  |  | 1 | 1.28 | 0.03594 | 2 | 1.14 | 0.00651 |
| GO:0071322 | cellular response to carbohydrate stimulus | 11 |  |  |  | 1 | 2 | 0.015 | 2 | 1.79 | 0.01181 |
| GO:0009756 | carbohydrate mediated signaling | 9 |  |  |  | 1 | 1.64 | 0.04433 | 2 | 1.47 | 0.03681 |
| GO:0016102 | diterpenoid biosynthetic process | 7 |  |  |  | 1 | 1.28 | 0.03594 |  |  |  |
| GO:0009631 | cold acclimation | 6 |  |  |  | 1 | 1.09 | 0.03633 |  |  |  |
| GO:0045088 | regulation of innate immune response | 76 |  |  |  |  |  |  | 21 | 12.37 | 0.00899 |
| GO:0048827 | phyllome development | 90 |  |  |  |  |  |  | 21 | 14.65 | 0.04208 |
| GO:0009627 | systemic acquired resistance | 59 |  |  |  |  |  |  | 16 | 9.61 | 0.02129 |
| GO:0060548 | negative regulation of cell death | 38 |  |  |  |  |  |  | 13 | 6.19 | 0.0267 |
| GO:0009696 | salicylic acid metabolic process | 33 |  |  |  |  |  |  | 13 | 5.37 | 0.03842 |
| GO:0009741 | response to brassinosteroid | 29 |  |  |  |  |  |  | 12 | 4.72 | 0.00761 |
| GO:0000165 | MAPK cascade | 39 |  |  |  |  |  |  | 12 | 6.35 | 0.04249 |
| GO:0008202 | steroid metabolic process | 26 |  |  |  |  |  |  | 10 | 4.23 | 0.00902 |
| GO:0010260 | organ senescence | 35 |  |  |  |  |  |  | 10 | 5.7 | 0.01131 |
| GO:0006694 | steroid biosynthetic process | 23 |  |  |  |  |  |  | 9 | 3.74 | 0.01353 |
| GO:0032879 | regulation of localization | 23 |  |  |  |  |  |  | 9 | 3.74 | 0.02098 |
| GO:0051174 | regulation of phosphorus metabolic process | 35 |  |  |  |  |  |  | 9 | 5.7 | 0.04113 |
| GO:0019220 | regulation of phosphate metabolic process | 35 |  |  |  |  |  |  | 9 | 5.7 | 0.04113 |
| GO:0014070 | response to organic cyclic compound | 21 |  |  |  |  |  |  | 8 | 3.42 | 0.00436 |
| GO:0010675 | regulation of cellular carbohydrate metabolic process | 10 |  |  |  |  |  |  | 6 | 1.63 | 0.00723 |
| GO:0009595 | detection of biotic stimulus | 18 |  |  |  |  |  |  | 6 | 2.93 | 0.0136 |
| GO:0051049 | regulation of transport | 16 |  |  |  |  |  |  | 6 | 2.61 | 0.02032 |
| GO:0016126 | sterol biosynthetic process | 15 |  |  |  |  |  |  | 6 | 2.44 | 0.03154 |
| GO:0009640 | photomorphogenesis | 24 |  |  |  |  |  |  | 6 | 3.91 | 0.0319 |
| GO:0010150 | leaf senescence | 26 |  |  |  |  |  |  | 6 | 4.23 | 0.03714 |
| GO:0016125 | sterol metabolic process | 16 |  |  |  |  |  |  | 6 | 2.61 | 0.04299 |
| GO:0015833 | peptide transport | 10 |  |  |  |  |  |  | 5 | 1.63 | 0.03458 |
| GO:0006857 | oligopeptide transport | 10 |  |  |  |  |  |  | 5 | 1.63 | 0.03458 |
| GO:0045089 | positive regulation of innate immune response | 11 |  |  |  |  |  |  | 5 | 1.79 | 0.0408 |
| GO:0002684 | positive regulation of immune system process | 11 |  |  |  |  |  |  | 5 | 1.79 | 0.0408 |
| GO:0050778 | positive regulation of immune response | 11 |  |  |  |  |  |  | 5 | 1.79 | 0.0408 |
| GO:0031349 | positive regulation of defense response | 11 |  |  |  |  |  |  | 5 | 1.79 | 0.0408 |
| GO:0009648 | photoperiodism | 17 |  |  |  |  |  |  | 5 | 2.77 | 0.04876 |
| GO:0048455 | stamen formation | 6 |  |  |  |  |  |  | 4 | 0.98 | 0.02666 |
| GO:0048448 | stamen morphogenesis | 6 |  |  |  |  |  |  | 4 | 0.98 | 0.02666 |
| GO:0048654 | anther morphogenesis | 6 |  |  |  |  |  |  | 4 | 0.98 | 0.02666 |
| GO:0048655 | anther wall tapetum morphogenesis | 6 |  |  |  |  |  |  | 4 | 0.98 | 0.02666 |
| GO:0048656 | anther wall tapetum formation | 6 |  |  |  |  |  |  | 4 | 0.98 | 0.02666 |
| GO:0048657 | anther wall tapetum cell differentiation | 6 |  |  |  |  |  |  | 4 | 0.98 | 0.02666 |
| GO:0048658 | anther wall tapetum development | 6 |  |  |  |  |  |  | 4 | 0.98 | 0.02666 |
| GO:0046490 | isopentenyl diphosphate metabolic process | 19 |  |  |  |  |  |  | 2 | 3.09 | 0.02288 |
| GO:0009240 | isopentenyl diphosphate biosynthetic process | 19 |  |  |  |  |  |  | 2 | 3.09 | 0.02288 |
| GO:0019682 | glyceraldehyde-3-phosphate metabolic process | 19 |  |  |  |  |  |  | 2 | 3.09 | 0.02288 |
| GO:0006090 | pyruvate metabolic process | 19 |  |  |  |  |  |  | 2 | 3.09 | 0.02288 |

Note:GO ID indicate the ID of GO term；Term indicate GO gunction；Annotated indicate the annotated genes numbers in all genes；Significant indicate the annotated DEGs number；Expected indicate the expect value for the annotated DEGs；KS indicate the significant statistics of enriched terms, the smaller KS value shows higher significanlt enrichment.
