# Supplementary material for: Identification of Key Gene Networks and Deciphering Transcriptional Regulators Associated With Peanut Embryo Abortion Mediated by Calcium Deficiency
Source: Front Plant Sci. 2022 Mar 21;13:814015. doi: 10.3389/fpls.2022.814015 (PMC8978587; doi:10.3389/fpls.2022.814015)
Supplement: Supplementary file 8 [file Table_4.docx]

**Supplementary Table 4 Molecular function TopGO enrichment results of DEGs**

| **GO ID** | **Term** | **Annotated** | **15DAP** | | | **20DAP** | |  | **30DAP** | | |
| --- | --- | --- | --- | --- | --- | --- | --- | --- | --- | --- | --- |
|  |  |  | **Significant** | **Expected** | **KS** | **Significant** | **Expected** | **KS** | **Significant** | **Expected** | **KS** |
| GO:0005516 | calmodulin binding | 10 | 0 | 0.2 | 0.013 | 6 | 1.79 | 0.00056 | 10 | 2.74 | 0.001 |
| GO:0019899 | enzyme binding | 20 | 0 | 0.4 | 0.014 | 10 | 3.23 | 0.0028 | 3 | 1.52 | 0.0032 |
| GO:0043169 | cation binding | 144 | 1 | 2.85 | 0.018 | 13 | 7.54 | 0.00537 | 13 | 6.39 | 0.0033 |
| GO:0042802 | identical protein binding | 42 | 1 | 0.83 | 0.024 | 4 | 1.26 | 0.02096 | 17 | 8.22 | 0.0041 |
| GO:0046872 | metal ion binding | 141 | 1 | 2.79 | 0.027 | 2 | 2.15 | 0.02352 | 5 | 1.07 | 0.0046 |
| GO:0043167 | ion binding | 149 | 1 | 2.95 | 0.039 | 6 | 1.79 | 0.02568 | 9 | 3.96 | 0.0144 |
| GO:0030234 | enzyme regulator activity | 13 | 0 | 0.26 | 0.058 | 14 | 9.69 | 0.02958 | 6 | 1.98 | 0.0209 |
| GO:0043565 | sequence-specific DNA binding | 50 | 3 | 0.99 | 0.06 | 5 | 1.62 | 0.03308 | 4 | 1.37 | 0.0236 |
| GO:0016709 | oxidoreductase activity, acting on paired donors, with incorporation or reduction of molecular oxygen, NAD(P)H as one donor, and incorporation of one atom of oxygen | 6 | 0 | 0.12 | 0.083 | 10 | 8.97 | 0.05577 | 18 | 13.4 | 0.0371 |
| GO:0030247 | polysaccharide binding | 7 | 0 | 0.14 | 0.097 | 1 | 1.62 | 0.0607 | 4 | 1.07 | 0.0442 |
| GO:0001871 | pattern binding | 7 | 0 | 0.14 | 0.097 | 2 | 1.62 | 0.0607 | 18 | 13.24 | 0.0453 |
| GO:0042803 | protein homodimerization activity | 26 | 0 | 0.51 | 0.108 | 21 | 26.74 | 0.07427 | 11 | 7.61 | 0.0586 |
| GO:0016881 | acid-amino acid ligase activity | 27 | 0 | 0.53 | 0.111 | 7 | 4.67 | 0.0759 | 4 | 1.98 | 0.0632 |
| GO:0004842 | ubiquitin-protein transferase activity | 27 | 0 | 0.53 | 0.111 | 18 | 25.84 | 0.08056 | 17 | 21.92 | 0.0674 |
| GO:0019787 | small conjugating protein transferase activity | 27 | 0 | 0.53 | 0.111 | 1 | 1.44 | 0.08313 | 6 | 2.13 | 0.0805 |
| GO:0004857 | enzyme inhibitor activity | 9 | 0 | 0.18 | 0.114 | 4 | 1.26 | 0.0836 | 6 | 2.13 | 0.0805 |
| GO:0016741 | transferase activity, transferring one-carbon groups | 10 | 0 | 0.2 | 0.115 | 5 | 2.33 | 0.08943 | 19 | 22.68 | 0.0919 |
| GO:0008168 | methyltransferase activity | 10 | 0 | 0.2 | 0.115 | 8 | 4.13 | 0.09464 | 16 | 21.46 | 0.0919 |
| GO:0004497 | monooxygenase activity | 9 | 0 | 0.18 | 0.117 | 8 | 4.13 | 0.09464 | 1 | 3.04 | 0.1006 |
| GO:0008509 | anion transmembrane transporter activity | 9 | 1 | 0.18 | 0.13 | 17 | 25.3 | 0.10757 | 6 | 2.89 | 0.1238 |
| GO:0001071 | nucleic acid binding transcription factor activity | 88 | 3 | 1.74 | 0.131 | 17 | 15.79 | 0.1103 | 3 | 1.22 | 0.1247 |
| GO:0016705 | oxidoreductase activity, acting on paired donors, with incorporation or reduction of molecular oxygen | 12 | 0 | 0.24 | 0.133 | 2 | 1.79 | 0.11361 | 3 | 1.22 | 0.1247 |
| GO:0008194 | UDP-glycosyltransferase activity | 10 | 0 | 0.2 | 0.141 | 2 | 1.79 | 0.11361 | 3 | 1.22 | 0.1247 |
| GO:0046914 | transition metal ion binding | 87 | 1 | 1.72 | 0.143 | 1 | 1.26 | 0.11375 | 3 | 3.81 | 0.1356 |
| GO:0005488 | binding | 592 | 13 | 11.72 | 0.145 | 6 | 2.51 | 0.1243 | 0 | 0.91 | 0.1381 |
| GO:0050661 | NADP binding | 12 | 0 | 0.24 | 0.155 | 6 | 2.51 | 0.1243 | 7 | 3.5 | 0.145 |
| GO:0042625 | ATPase activity, coupled to transmembrane movement of ions | 14 | 0 | 0.28 | 0.156 | 7 | 3.59 | 0.12481 | 7 | 3.5 | 0.145 |
| GO:0016879 | ligase activity, forming carbon-nitrogen bonds | 30 | 0 | 0.59 | 0.157 | 17 | 15.61 | 0.12922 | 7 | 1.52 | 0.1611 |
| GO:0003700 | sequence-specific DNA binding transcription factor activity | 87 | 3 | 1.72 | 0.162 | 1 | 3.59 | 0.13003 | 2 | 1.07 | 0.1902 |
| GO:0046983 | protein dimerization activity | 54 | 1 | 1.07 | 0.163 | 4 | 1.44 | 0.14164 | 2 | 1.07 | 0.1902 |
| GO:0004553 | hydrolase activity, hydrolyzing O-glycosyl compounds | 24 | 0 | 0.48 | 0.165 | 2 | 1.62 | 0.14348 | 3 | 4.11 | 0.1953 |
| GO:0016874 | ligase activity | 43 | 0 | 0.85 | 0.167 | 2 | 1.44 | 0.14562 | 3 | 4.11 | 0.1953 |
| GO:0046906 | tetrapyrrole binding | 8 | 0 | 0.16 | 0.174 | 2 | 1.44 | 0.14562 | 3 | 4.11 | 0.1953 |
| GO:0044212 | transcription regulatory region DNA binding | 8 | 0 | 0.16 | 0.18 | 2 | 1.44 | 0.14562 | 2 | 2.28 | 0.1979 |
| GO:0001067 | regulatory region nucleic acid binding | 8 | 0 | 0.16 | 0.18 | 5 | 4.49 | 0.14976 | 3 | 4.41 | 0.205 |
| GO:0000975 | regulatory region DNA binding | 8 | 0 | 0.16 | 0.18 | 0 | 1.08 | 0.15554 | 3 | 1.07 | 0.2059 |
| GO:0015103 | inorganic anion transmembrane transporter activity | 6 | 1 | 0.12 | 0.185 | 3 | 2.69 | 0.16837 | 1 | 1.37 | 0.207 |
| GO:0015075 | ion transmembrane transporter activity | 29 | 1 | 0.57 | 0.188 | 4 | 4.85 | 0.16865 | 1 | 1.37 | 0.2083 |
| GO:0070696 | transmembrane receptor protein serine/threonine kinase binding | 7 | 0 | 0.14 | 0.197 | 4 | 4.85 | 0.16865 | 1 | 1.37 | 0.2083 |
| GO:0004872 | receptor activity | 19 | 2 | 0.38 | 0.211 | 4 | 4.85 | 0.16865 | 2 | 1.67 | 0.2293 |
| GO:0003676 | nucleic acid binding | 211 | 4 | 4.18 | 0.231 | 6 | 3.41 | 0.18147 | 5 | 2.74 | 0.2309 |
| GO:0051287 | NAD binding | 7 | 1 | 0.14 | 0.239 | 5 | 5.2 | 0.18158 | 4 | 5.63 | 0.2367 |
| GO:0019829 | cation-transporting ATPase activity | 9 | 0 | 0.18 | 0.242 | 3 | 2.33 | 0.18484 | 12 | 13.24 | 0.2387 |
| GO:0005509 | calcium ion binding | 9 | 0 | 0.18 | 0.247 | 2 | 1.26 | 0.21394 | 2 | 1.98 | 0.2465 |
| GO:0016301 | kinase activity | 90 | 3 | 1.78 | 0.252 | 2 | 1.26 | 0.21394 | 0 | 1.83 | 0.2515 |
| GO:0020037 | heme binding | 7 | 0 | 0.14 | 0.254 | 2 | 2.15 | 0.21487 | 3 | 3.65 | 0.26 |
| GO:0016758 | transferase activity, transferring hexosyl groups | 20 | 0 | 0.4 | 0.254 | 7 | 4.13 | 0.21687 | 3 | 4.57 | 0.2695 |
| GO:0033612 | receptor serine/threonine kinase binding | 14 | 1 | 0.28 | 0.259 | 4 | 5.38 | 0.21833 | 3 | 1.83 | 0.2712 |
| GO:0005102 | receptor binding | 14 | 1 | 0.28 | 0.259 | 3 | 2.33 | 0.22869 | 2 | 1.07 | 0.273 |
| GO:0008324 | cation transmembrane transporter activity | 25 | 1 | 0.5 | 0.267 | 3 | 1.62 | 0.2448 | 1 | 1.52 | 0.2821 |
| GO:0004175 | endopeptidase activity | 9 | 1 | 0.18 | 0.269 | 24 | 25.3 | 0.24939 | 1 | 1.52 | 0.2821 |
| GO:0004722 | protein serine/threonine phosphatase activity | 11 | 0 | 0.22 | 0.272 | 14 | 15.61 | 0.25769 | 0 | 1.22 | 0.2824 |
| GO:0070011 | peptidase activity, acting on L-amino acid peptides | 16 | 1 | 0.32 | 0.274 | 2 | 2.51 | 0.26274 | 0 | 1.83 | 0.2853 |
| GO:0016757 | transferase activity, transferring glycosyl groups | 23 | 0 | 0.46 | 0.281 | 2 | 1.97 | 0.27412 | 25 | 21.46 | 0.2946 |
| GO:0008233 | peptidase activity | 24 | 1 | 0.48 | 0.293 | 4 | 4.31 | 0.28155 | 13 | 13.7 | 0.2985 |
| GO:0042626 | ATPase activity, coupled to transmembrane movement of substances | 29 | 0 | 0.57 | 0.295 | 7 | 4.13 | 0.29744 | 1 | 1.67 | 0.3067 |
| GO:0043492 | ATPase activity, coupled to movement of substances | 29 | 0 | 0.57 | 0.295 | 2 | 1.97 | 0.30001 | 7 | 3.04 | 0.3214 |
| GO:0016798 | hydrolase activity, acting on glycosyl bonds | 31 | 0 | 0.61 | 0.304 | 3 | 1.26 | 0.30592 | 88 | 90.12 | 0.3252 |
| GO:0008270 | zinc ion binding | 65 | 0 | 1.29 | 0.309 | 5 | 3.23 | 0.31447 | 1 | 1.37 | 0.3254 |
| GO:0019199 | transmembrane receptor protein kinase activity | 10 | 1 | 0.2 | 0.31 | 10 | 15.61 | 0.31588 | 2 | 1.07 | 0.3415 |
| GO:0022890 | inorganic cation transmembrane transporter activity | 15 | 0 | 0.3 | 0.323 | 11 | 8.25 | 0.31871 | 5 | 3.5 | 0.3458 |
| GO:0005515 | protein binding | 247 | 8 | 4.89 | 0.326 | 0 | 2.15 | 0.32171 | 3 | 1.37 | 0.3527 |
| GO:0016860 | intramolecular oxidoreductase activity | 7 | 2 | 0.14 | 0.328 | 3 | 1.26 | 0.33219 | 3 | 6.55 | 0.3534 |
| GO:0046982 | protein heterodimerization activity | 13 | 0 | 0.26 | 0.331 | 3 | 1.44 | 0.3498 | 9 | 7.46 | 0.3564 |
| GO:0003723 | RNA binding | 37 | 0 | 0.73 | 0.331 | 3 | 1.26 | 0.35005 | 2 | 1.52 | 0.3628 |
| GO:0003677 | DNA binding | 141 | 3 | 2.79 | 0.337 | 36 | 37.87 | 0.35548 | 32 | 32.12 | 0.366 |
| GO:0016491 | oxidoreductase activity | 87 | 1 | 1.72 | 0.349 | 1 | 1.62 | 0.36678 | 0 | 1.07 | 0.3797 |
| GO:0009055 | electron carrier activity | 12 | 0 | 0.24 | 0.35 | 3 | 2.15 | 0.37709 | 3 | 3.2 | 0.3828 |
| GO:0050660 | flavin adenine dinucleotide binding | 7 | 0 | 0.14 | 0.367 | 7 | 5.56 | 0.39292 | 3 | 1.52 | 0.3893 |
| GO:0016820 | hydrolase activity, acting on acid anhydrides, catalyzing transmembrane movement of substances | 30 | 0 | 0.59 | 0.37 | 5 | 7.72 | 0.39718 | 1 | 0.91 | 0.3894 |
| GO:0016773 | phosphotransferase activity, alcohol group as acceptor | 74 | 3 | 1.47 | 0.371 | 3 | 1.26 | 0.39832 | 8 | 3.5 | 0.3901 |
| GO:0005506 | iron ion binding | 9 | 0 | 0.18 | 0.375 | 17 | 16.15 | 0.39924 | 0 | 2.13 | 0.3973 |
| GO:0038023 | signaling receptor activity | 18 | 2 | 0.36 | 0.377 | 2 | 1.79 | 0.4132 | 32 | 29.23 | 4.48E-01 |
| GO:0050662 | coenzyme binding | 32 | 1 | 0.63 | 0.383 | 4 | 5.2 | 0.42176 | 5 | 4.72 | 0.4499 |
| GO:0015078 | hydrogen ion transmembrane transporter activity | 11 | 0 | 0.22 | 0.411 | 4 | 5.2 | 0.42176 | 1 | 1.83 | 0.4679 |
| GO:0016772 | transferase activity, transferring phosphorus-containing groups | 114 | 4 | 2.26 | 0.419 | 1 | 1.08 | 0.42599 | 3 | 1.52 | 0.4683 |
| GO:0004672 | protein kinase activity | 62 | 3 | 1.23 | 0.427 | 6 | 7.18 | 0.42741 | 0 | 1.37 | 0.4695 |
| GO:0005507 | copper ion binding | 12 | 1 | 0.24 | 0.435 | 3 | 1.79 | 0.43243 | 1 | 0.91 | 0.4732 |
| GO:0016740 | transferase activity | 192 | 5 | 3.8 | 0.44 | 7 | 5.56 | 0.4579 | 8 | 7 | 0.4874 |
| GO:0004518 | nuclease activity | 12 | 0 | 0.24 | 0.442 | 5 | 5.74 | 0.46798 | 9 | 11.26 | 0.5182 |
| GO:0004721 | phosphoprotein phosphatase activity | 15 | 0 | 0.3 | 0.442 | 108 | 106.24 | 0.47687 | 7 | 4.72 | 0.5188 |
| GO:0030246 | carbohydrate binding | 23 | 1 | 0.46 | 0.444 | 1 | 1.08 | 0.49096 | 16 | 17.35 | 0.5352 |
| GO:0043225 | anion transmembrane-transporting ATPase activity | 7 | 0 | 0.14 | 0.444 | 6 | 6.64 | 0.49178 | 3 | 2.28 | 0.55 |
| GO:0035251 | UDP-glucosyltransferase activity | 7 | 0 | 0.14 | 0.448 | 13 | 11.13 | 0.49363 | 3 | 4.87 | 0.561 |
| GO:0016779 | nucleotidyltransferase activity | 21 | 1 | 0.42 | 0.449 | 2 | 1.26 | 0.50003 | 3 | 4.87 | 0.561 |
| GO:0016853 | isomerase activity | 17 | 2 | 0.34 | 0.451 | 9 | 8.79 | 0.516 | 4 | 4.87 | 0.5783 |
| GO:0004888 | transmembrane signaling receptor activity | 13 | 1 | 0.26 | 0.46 | 4 | 5.38 | 0.5269 | 0 | 1.22 | 0.59 |
| GO:0004674 | protein serine/threonine kinase activity | 46 | 3 | 0.91 | 0.481 | 22 | 20.46 | 0.54209 | 0 | 0.91 | 0.5966 |
| GO:0048037 | cofactor binding | 40 | 1 | 0.79 | 0.486 | 3 | 1.62 | 0.55493 | 0 | 0.91 | 0.5966 |
| GO:0003779 | actin binding | 8 | 0 | 0.16 | 0.501 | 2 | 2.69 | 0.55582 | 1 | 4.41 | 0.6043 |
| GO:0008378 | galactosyltransferase activity | 6 | 0 | 0.12 | 0.52 | 4 | 3.05 | 0.57128 | 1 | 4.41 | 0.6043 |
| GO:0047274 | galactinol-sucrose galactosyltransferase activity | 6 | 0 | 0.12 | 0.52 | 5 | 3.77 | 0.57805 | 2 | 1.98 | 0.6073 |
| GO:0016614 | oxidoreductase activity, acting on CH-OH group of donors | 32 | 1 | 0.63 | 0.52 | 36 | 34.46 | 0.58765 | 3 | 1.83 | 0.6087 |
| GO:0016616 | oxidoreductase activity, acting on the CH-OH group of donors, NAD or NADP as acceptor | 32 | 1 | 0.63 | 0.52 | 0 | 1.44 | 0.60707 | 12 | 13.24 | 0.6315 |
| GO:0015077 | monovalent inorganic cation transmembrane transporter activity | 13 | 0 | 0.26 | 0.523 | 1 | 1.08 | 0.6114 | 2 | 1.07 | 0.6351 |
| GO:0022804 | active transmembrane transporter activity | 45 | 1 | 0.89 | 0.531 | 1 | 1.08 | 0.6114 | 0 | 1.07 | 0.6459 |
| GO:0042578 | phosphoric ester hydrolase activity | 22 | 0 | 0.44 | 0.531 | 3 | 1.79 | 0.61495 | 1 | 1.37 | 0.6509 |
| GO:1901265 | nucleoside phosphate binding | 169 | 6 | 3.35 | 0.537 | 13 | 13.28 | 0.65192 | 8 | 9.89 | 0.6543 |
| GO:0000166 | nucleotide binding | 169 | 6 | 3.35 | 0.537 | 4 | 3.95 | 0.67533 | 2 | 2.59 | 0.6682 |
| GO:0004871 | signal transducer activity | 23 | 2 | 0.46 | 0.538 | 2 | 1.08 | 0.68356 | 4 | 3.35 | 0.6683 |
| GO:0060089 | molecular transducer activity | 23 | 2 | 0.46 | 0.538 | 2 | 1.08 | 0.68356 | 8 | 9.44 | 0.6852 |
| GO:0015405 | P-P-bond-hydrolysis-driven transmembrane transporter activity | 35 | 0 | 0.69 | 0.566 | 3 | 2.15 | 0.68513 | 2 | 1.22 | 0.6876 |
| GO:0015399 | primary active transmembrane transporter activity | 35 | 0 | 0.69 | 0.566 | 0 | 1.26 | 0.68542 | 2 | 1.67 | 0.6964 |
| GO:0008047 | enzyme activator activity | 7 | 0 | 0.14 | 0.575 | 4 | 5.74 | 0.70306 | 1 | 4.57 | 0.7092 |
| GO:0032561 | guanyl ribonucleotide binding | 10 | 0 | 0.2 | 0.579 | 4 | 5.74 | 0.70306 | 3 | 3.04 | 0.7181 |
| GO:0005525 | GTP binding | 10 | 0 | 0.2 | 0.579 | 5 | 11.66 | 0.7162 | 0 | 1.07 | 0.7182 |
| GO:0019001 | guanyl nucleotide binding | 10 | 0 | 0.2 | 0.579 | 1 | 1.97 | 0.72029 | 1 | 1.67 | 0.7203 |
| GO:0016791 | phosphatase activity | 20 | 0 | 0.4 | 0.59 | 86 | 86.86 | 0.72435 | 1 | 2.44 | 0.7348 |
| GO:0008092 | cytoskeletal protein binding | 14 | 0 | 0.28 | 0.62 | 3 | 3.59 | 0.72465 | 6 | 6.09 | 0.743 |
| GO:0015291 | secondary active transmembrane transporter activity | 11 | 1 | 0.22 | 0.631 | 2 | 1.62 | 0.72624 | 0 | 1.07 | 0.7602 |
| GO:0003682 | chromatin binding | 12 | 0 | 0.24 | 0.645 | 6 | 7.36 | 0.74202 | 19 | 26.64 | 0.7605 |
| GO:0003774 | motor activity | 8 | 0 | 0.16 | 0.645 | 3 | 4.31 | 0.75895 | 0 | 1.52 | 0.7638 |
| GO:0005543 | phospholipid binding | 6 | 0 | 0.12 | 0.696 | 0 | 1.26 | 0.75971 | 0 | 1.52 | 0.7638 |
| GO:0035091 | phosphatidylinositol binding | 6 | 0 | 0.12 | 0.696 | 28 | 31.4 | 0.76054 | 0 | 1.52 | 0.7638 |
| GO:0022891 | substrate-specific transmembrane transporter activity | 48 | 1 | 0.95 | 0.698 | 27 | 30.33 | 0.76962 | 1 | 0.91 | 0.7659 |
| GO:0003824 | catalytic activity | 484 | 10 | 9.58 | 0.701 | 27 | 30.33 | 0.76962 | 1 | 0.91 | 0.7659 |
| GO:0004519 | endonuclease activity | 11 | 0 | 0.22 | 0.701 | 5 | 8.08 | 0.78239 | 1 | 1.83 | 0.7685 |
| GO:0016830 | carbon-carbon lyase activity | 6 | 0 | 0.12 | 0.702 | 2 | 2.33 | 0.78362 | 18 | 25.73 | 0.7696 |
| GO:0016831 | carboxy-lyase activity | 6 | 0 | 0.12 | 0.702 | 0 | 1.79 | 0.78561 | 18 | 25.73 | 0.7696 |
| GO:0016462 | pyrophosphatase activity | 68 | 1 | 1.35 | 0.716 | 0 | 1.79 | 0.78561 | 2 | 2.13 | 0.7742 |
| GO:0036094 | small molecule binding | 175 | 6 | 3.47 | 0.722 | 0 | 1.79 | 0.78561 | 11 | 18.42 | 0.794 |
| GO:0042623 | ATPase activity, coupled | 41 | 0 | 0.81 | 0.726 | 0 | 1.26 | 0.78935 | 1 | 0.91 | 0.8003 |
| GO:0022857 | transmembrane transporter activity | 59 | 1 | 1.17 | 0.726 | 19 | 21.71 | 0.79404 | 1 | 0.91 | 0.8003 |
| GO:0005215 | transporter activity | 66 | 1 | 1.31 | 0.732 | 4 | 6.28 | 0.80924 | 2 | 6.85 | 0.8009 |
| GO:0016628 | oxidoreductase activity, acting on the CH-CH group of donors, NAD or NADP as acceptor | 8 | 0 | 0.16 | 0.737 | 4 | 6.28 | 0.80924 | 11 | 16.9 | 0.8118 |
| GO:0046527 | glucosyltransferase activity | 8 | 0 | 0.16 | 0.737 | 19 | 19.92 | 0.81178 | 4 | 7.92 | 0.8147 |
| GO:0016651 | oxidoreductase activity, acting on NAD(P)H | 7 | 0 | 0.14 | 0.751 | 1 | 1.44 | 0.81467 | 2 | 3.65 | 0.8147 |
| GO:0044389 | small conjugating protein ligase binding | 8 | 0 | 0.16 | 0.753 | 1 | 1.44 | 0.81467 | 1 | 1.07 | 0.8158 |
| GO:0031625 | ubiquitin protein ligase binding | 8 | 0 | 0.16 | 0.753 | 3 | 2.15 | 0.81967 | 1 | 1.22 | 0.8183 |
| GO:0008080 | N-acetyltransferase activity | 12 | 0 | 0.24 | 0.754 | 3 | 2.87 | 0.82256 | 1 | 1.22 | 0.8183 |
| GO:0003924 | GTPase activity | 6 | 0 | 0.12 | 0.765 | 19 | 22.43 | 0.83427 | 11 | 19.03 | 0.8343 |
| GO:0032440 | 2-alkenal reductase [NAD(P)] activity | 7 | 0 | 0.14 | 0.766 | 19 | 22.07 | 0.83712 | 11 | 18.72 | 0.8371 |
| GO:0016817 | hydrolase activity, acting on acid anhydrides | 72 | 1 | 1.43 | 0.77 | 19 | 22.07 | 0.83712 | 11 | 18.72 | 0.8371 |
| GO:0016818 | hydrolase activity, acting on acid anhydrides, in phosphorus-containing anhydrides | 69 | 1 | 1.37 | 0.777 | 29 | 32.84 | 0.83859 | 19 | 27.86 | 0.8386 |
| GO:0003735 | structural constituent of ribosome | 27 | 0 | 0.53 | 0.779 | 4 | 4.31 | 0.83981 | 1 | 5.33 | 0.8433 |
| GO:0097159 | organic cyclic compound binding | 183 | 6 | 3.62 | 0.788 | 19 | 20.64 | 0.84849 | 1 | 5.33 | 0.8433 |
| GO:0035639 | purine ribonucleoside triphosphate binding | 121 | 4 | 2.4 | 0.789 | 19 | 20.28 | 0.85131 | 11 | 17.51 | 0.8485 |
| GO:0004713 | protein tyrosine kinase activity | 9 | 1 | 0.18 | 0.791 | 6 | 9.33 | 0.85182 | 0 | 1.22 | 0.8501 |
| GO:0022892 | substrate-specific transporter activity | 52 | 1 | 1.03 | 0.797 | 7 | 10.59 | 0.8647 | 0 | 0.91 | 0.851 |
| GO:0016788 | hydrolase activity, acting on ester bonds | 49 | 0 | 0.97 | 0.804 | 2 | 2.51 | 0.86532 | 11 | 17.2 | 0.8513 |
| GO:0005524 | ATP binding | 111 | 4 | 2.2 | 0.816 | 0 | 1.08 | 0.87385 | 2 | 2.28 | 0.8574 |
| GO:0016887 | ATPase activity | 46 | 0 | 0.91 | 0.82 | 33 | 35.17 | 0.87462 | 1 | 1.67 | 0.8631 |
| GO:0004402 | histone acetyltransferase activity | 8 | 0 | 0.16 | 0.822 | 2 | 1.44 | 0.87642 | 4 | 8.98 | 0.8675 |
| GO:0017111 | nucleoside-triphosphatase activity | 64 | 0 | 1.27 | 0.826 | 2 | 1.44 | 0.87642 | 4 | 3.65 | 0.8786 |
| GO:0005198 | structural molecule activity | 34 | 0 | 0.67 | 0.848 | 9 | 12.92 | 0.87977 | 5 | 10.96 | 0.8798 |
| GO:0016829 | lyase activity | 11 | 0 | 0.22 | 0.85 | 9 | 11.84 | 0.88089 | 6 | 10.05 | 0.8809 |
| GO:0017076 | purine nucleotide binding | 125 | 4 | 2.48 | 0.856 | 0 | 1.44 | 0.88269 | 1 | 1.83 | 0.8846 |
| GO:0032559 | adenyl ribonucleotide binding | 113 | 4 | 2.24 | 0.857 | 9 | 12.38 | 0.88492 | 5 | 10.5 | 0.8849 |
| GO:0032553 | ribonucleotide binding | 123 | 4 | 2.44 | 0.86 | 9 | 12.2 | 0.88663 | 5 | 10.35 | 0.8866 |
| GO:0032555 | purine ribonucleotide binding | 123 | 4 | 2.44 | 0.86 | 52 | 44.33 | 0.88736 | 3 | 7.31 | 0.8923 |
| GO:0030554 | adenyl nucleotide binding | 115 | 4 | 2.28 | 0.861 | 2 | 1.97 | 0.88788 | 3 | 9.74 | 0.8935 |
| GO:0046873 | metal ion transmembrane transporter activity | 6 | 0 | 0.12 | 0.863 | 5 | 8.61 | 0.89229 | 5 | 1.22 | 0.8945 |
| GO:0016410 | N-acyltransferase activity | 14 | 0 | 0.28 | 0.863 | 7 | 11.49 | 0.89347 | 1 | 1.07 | 0.8983 |
| GO:0034061 | DNA polymerase activity | 12 | 0 | 0.24 | 0.879 | 1 | 1.62 | 0.91047 | 0 | 1.83 | 0.9198 |
| GO:0000287 | magnesium ion binding | 8 | 0 | 0.16 | 0.889 | 1 | 1.44 | 0.91744 | 1 | 1.37 | 0.9219 |
| GO:0003690 | double-stranded DNA binding | 7 | 0 | 0.14 | 0.892 | 6 | 8.25 | 0.924 | 2 | 7 | 0.924 |
| GO:0008289 | lipid binding | 18 | 1 | 0.36 | 0.892 | 1 | 2.15 | 0.93031 | 1 | 1.22 | 0.9253 |
| GO:0016407 | acetyltransferase activity | 15 | 0 | 0.3 | 0.903 | 1 | 1.97 | 0.936 | 2 | 6.24 | 0.9275 |
| GO:0003964 | RNA-directed DNA polymerase activity | 11 | 0 | 0.22 | 0.908 | 0 | 1.08 | 0.93757 | 1 | 4.11 | 0.9356 |
| GO:0016746 | transferase activity, transferring acyl groups | 31 | 1 | 0.61 | 0.909 | 0 | 1.08 | 0.93757 | 0 | 1.22 | 0.9446 |
| GO:0052689 | carboxylic ester hydrolase activity | 10 | 0 | 0.2 | 0.911 | 1 | 2.15 | 0.94664 | 1 | 1.07 | 0.9592 |
| GO:0016903 | oxidoreductase activity, acting on the aldehyde or oxo group of donors | 12 | 0 | 0.24 | 0.928 | 1 | 1.26 | 0.96429 | 43 | 37.6 | 0.96 |
| GO:0016620 | oxidoreductase activity, acting on the aldehyde or oxo group of donors, NAD or NADP as acceptor | 12 | 0 | 0.24 | 0.928 | 0 | 1.44 | 0.96489 | 25 | 29.84 | 0.961 |
| GO:0016787 | hydrolase activity | 196 | 2 | 3.88 | 0.933 | 1 | 2.69 | 0.96644 | 0 | 0.91 | 0.9722 |
| GO:0016747 | transferase activity, transferring acyl groups other than amino-acyl groups | 24 | 1 | 0.48 | 0.946 | 0 | 2.15 | 0.9684 | 0 | 0.91 | 0.9722 |
| GO:0008565 | protein transporter activity | 7 | 0 | 0.14 | 0.951 | 0 | 1.08 | 0.96935 | 0 | 0.91 | 0.9722 |
| GO:0016627 | oxidoreductase activity, acting on the CH-CH group of donors | 10 | 0 | 0.2 | 0.956 | 0 | 1.08 | 0.96935 | 70 | 73.68 | 0.9732 |
| GO:0008320 | protein transmembrane transporter activity | 6 | 0 | 0.12 | 0.958 | 0 | 1.08 | 0.96935 | 2 | 1.83 | 0.9763 |
| GO:0022884 | macromolecule transmembrane transporter activity | 6 | 0 | 0.12 | 0.958 | 1 | 4.85 | 0.97299 | 0 | 2.13 | 0.9817 |
| GO:0015450 | P-P-bond-hydrolysis-driven protein transmembrane transporter activity | 6 | 0 | 0.12 | 0.958 | 1 | 1.26 | 0.98318 | 1 | 1.22 | 0.987 |
| GO:0004386 | helicase activity | 12 | 0 | 0.24 | 0.967 | 1 | 6.1 | 0.98693 | 1 | 1.22 | 0.987 |
| GO:0019843 | rRNA binding | 7 | 0 | 0.14 | 0.974 | 0 | 2.51 | 0.99023 | 1 | 5.18 | 0.9892 |
| GO:0008026 | ATP-dependent helicase activity | 8 | 0 | 0.16 | 0.978 | 1 | 2.15 | 0.99761 | 1 | 1.83 | 0.9927 |
| GO:0070035 | purine NTP-dependent helicase activity | 8 | 0 | 0.16 | 0.978 | 1 | 2.15 | 0.99761 | 1 | 1.83 | 0.9927 |
| GO:0043566 | structure-specific DNA binding | 9 | 0 | 0.18 | 0.994 | 0 | 1.97 | 0.99945 | 0 | 1.67 | 0.9997 |
| GO:0003674 | molecular_function | 808 | 16 | 16 | 1 | 145 | 145 | 1 | 123 | 123 | 1 |

Note:GO ID indicate the ID of GO term；Term indicate GO gunction；Annotated indicate the annotated genes numbers in all genes；Significant indicate the annotated DEGs number；Expected indicate the expect value for the annotated DEGs；KS indicate the significant statistics of enriched terms, the smaller KS value shows higher significanlt enrichment.
