# Supplementary material for: Identification of Key Gene Networks and Deciphering Transcriptional Regulators Associated With Peanut Embryo Abortion Mediated by Calcium Deficiency
Source: Front Plant Sci. 2022 Mar 21;13:814015. doi: 10.3389/fpls.2022.814015 (PMC8978587; doi:10.3389/fpls.2022.814015)
Supplement: Supplementary file 9 [file Table_5.docx]

**Supplementary Table 5 Cellular components TopGO enrichment results of DEGs**

| **GO ID** | **Term** | **Annotated** | **15DAP** | | | **20DAP** | |  | **30DAP** | | |
| --- | --- | --- | --- | --- | --- | --- | --- | --- | --- | --- | --- |
|  |  |  | **Significant** | **Expected** | **KS** | **Significant** | **Expected** | **KS** | **Significant** | **Expected** | **KS** |
| GO:0016607 | nuclear speck | 6 | 0 | 0.13 | 0.0078 | 3 | 1.08 | 0.0071 | 1 | 0.91 | 0.0047 |
| GO:0030427 | site of polarized growth | 10 | 2 | 0.21 | 0.0202 | 2 | 1.8 | 0.0688 | 1 | 1.51 | 0.1889 |
| GO:0005789 | endoplasmic reticulum membrane | 12 | 0 | 0.25 | 0.0236 | 0 | 2.16 | 0.0718 | 0 | 1.82 | 0.0599 |
| GO:0042175 | nuclear outer membrane-endoplasmic reticulum membrane network | 12 | 0 | 0.25 | 0.0236 | 0 | 2.16 | 0.0718 | 0 | 1.82 | 0.0599 |
| GO:0044432 | endoplasmic reticulum part | 12 | 0 | 0.25 | 0.0236 | 0 | 2.16 | 0.0718 | 0 | 1.82 | 0.0599 |
| GO:0090406 | pollen tube | 10 | 2 | 0.21 | 0.0239 | 3 | 1.8 | 0.0688 | 2 | 1.51 | 0.1889 |
| GO:0045259 | proton-transporting ATP synthase complex | 6 | 0 | 0.13 | 0.0278 | 2 | 1.08 | 0.0041 | 1 | 0.91 | 0.0094 |
| GO:0035838 | growing cell tip | 9 | 2 | 0.19 | 0.0349 | 2 | 1.62 | 0.0902 | 1 | 1.36 | 0.2857 |
| GO:0051286 | cell tip | 9 | 2 | 0.19 | 0.0349 | 2 | 1.62 | 0.0902 | 1 | 1.36 | 0.2857 |
| GO:0060187 | cell pole | 9 | 2 | 0.19 | 0.0349 | 2 | 1.62 | 0.0902 | 1 | 1.36 | 0.2857 |
| GO:0090404 | pollen tube tip | 9 | 2 | 0.19 | 0.0349 | 2 | 1.62 | 0.0902 | 1 | 1.36 | 0.2857 |
| GO:0042995 | cell projection | 11 | 2 | 0.23 | 0.0419 | 3 | 1.98 | 0.1091 | 2 | 1.67 | 0.2906 |
| GO:0019866 | organelle inner membrane | 22 | 0 | 0.46 | 0.0446 | 3 | 3.96 | 0.0356 | 3 | 3.33 | 0.0493 |
| GO:0044421 | extracellular region part | 6 | 1 | 0.13 | 0.0519 | 2 | 1.08 | 0.1529 | 0 | 0.91 | 0.488 |
| GO:0044463 | cell projection part | 10 | 2 | 0.21 | 0.0561 | 2 | 1.8 | 0.1338 | 1 | 1.51 | 0.3545 |
| GO:0005829 | cytosol | 171 | 6 | 3.61 | 0.071 | 33 | 30.76 | 0.3882 | 23 | 25.88 | 0.6193 |
| GO:0016469 | proton-transporting two-sector ATPase complex | 7 | 0 | 0.15 | 0.0778 | 2 | 1.26 | 0.9164 | 1 | 1.06 | 0.9116 |
| GO:0005634 | nucleus | 323 | 7 | 6.81 | 0.0779 | 72 | 58.11 | 0.0085 | 57 | 48.89 | 0.0773 |
| GO:0005622 | intracellular | 724 | 14 | 15.27 | 0.0795 | 128 | 130.25 | 0.3876 | 108 | 109.59 | 0.364 |
| GO:0000145 | exocyst | 7 | 1 | 0.15 | 0.0886 | 3 | 1.26 | 0.2126 | 0 | 1.06 | 0.5584 |
| GO:0005938 | cell cortex | 7 | 1 | 0.15 | 0.0886 | 3 | 1.26 | 0.2126 | 0 | 1.06 | 0.5584 |
| GO:0044448 | cell cortex part | 7 | 1 | 0.15 | 0.0886 | 3 | 1.26 | 0.2126 | 0 | 1.06 | 0.5584 |
| GO:0044424 | intracellular part | 723 | 14 | 15.25 | 0.0916 | 128 | 130.07 | 0.4189 | 108 | 109.44 | 0.3521 |
| GO:0044422 | organelle part | 224 | 2 | 4.72 | 0.1235 | 30 | 40.3 | 0.4638 | 19 | 33.91 | 0.7094 |
| GO:0009526 | plastid envelope | 48 | 0 | 1.01 | 0.1321 | 6 | 8.64 | 0.3724 | 0 | 7.27 | 0.4478 |
| GO:0009505 | plant-type cell wall | 29 | 0 | 0.61 | 0.1357 | 12 | 5.22 | 0.0312 | 10 | 4.39 | 0.0712 |
| GO:0005911 | cell-cell junction | 105 | 5 | 2.21 | 0.1476 | 32 | 18.89 | 1 | 27 | 15.89 | 1 |
| GO:0009506 | plasmodesma | 105 | 5 | 2.21 | 0.1476 | 32 | 18.89 | 0.0013 | 27 | 15.89 | 0.0038 |
| GO:0030054 | cell junction | 105 | 5 | 2.21 | 0.1476 | 32 | 18.89 | 1 | 27 | 15.89 | 1 |
| GO:0055044 | symplast | 105 | 5 | 2.21 | 0.1476 | 32 | 18.89 | 1 | 27 | 15.89 | 1 |
| GO:0044446 | intracellular organelle part | 223 | 2 | 4.7 | 0.152 | 30 | 40.12 | 0.516 | 19 | 33.75 | 0.7241 |
| GO:0005618 | cell wall | 60 | 2 | 1.27 | 0.1561 | 22 | 10.79 | 0.0069 | 16 | 9.08 | 0.059 |
| GO:0042170 | plastid membrane | 8 | 0 | 0.17 | 0.1609 | 0 | 1.44 | 0.2807 | 0 | 1.21 | 0.2531 |
| GO:0031224 | intrinsic component of membrane | 76 | 2 | 1.6 | 0.1618 | 12 | 13.67 | 0.1999 | 12 | 11.5 | 0.1447 |
| GO:0030312 | external encapsulating structure | 62 | 2 | 1.31 | 0.1672 | 22 | 11.15 | 0.9592 | 16 | 9.38 | 0.0819 |
| GO:0015629 | actin cytoskeleton | 6 | 0 | 0.13 | 0.1711 | 0 | 1.08 | 0.2897 | 0 | 0.91 | 0.2653 |
| GO:0045177 | apical part of cell | 7 | 1 | 0.15 | 0.1717 | 0 | 1.26 | 0.3985 | 1 | 1.06 | 0.2779 |
| GO:0031090 | organelle membrane | 90 | 0 | 1.9 | 0.1786 | 10 | 16.19 | 0.4671 | 5 | 13.62 | 0.6374 |
| GO:0009941 | chloroplast envelope | 46 | 0 | 0.97 | 0.1925 | 6 | 8.28 | 0.3824 | 0 | 6.96 | 0.5537 |
| GO:0031981 | nuclear lumen | 55 | 1 | 1.16 | 0.1964 | 12 | 9.89 | 0.2329 | 7 | 8.33 | 0.7022 |
| GO:0044435 | plastid part | 78 | 1 | 1.65 | 0.2039 | 9 | 14.03 | 0.5558 | 5 | 11.81 | 0.4344 |
| GO:0009579 | thylakoid | 25 | 0 | 0.53 | 0.2102 | 6 | 4.5 | 0.1453 | 4 | 3.78 | 0.1039 |
| GO:0010319 | stromule | 9 | 0 | 0.19 | 0.2115 | 0 | 1.62 | 0.3855 | 0 | 1.36 | 0.3504 |
| GO:0031969 | chloroplast membrane | 6 | 0 | 0.13 | 0.2136 | 0 | 1.08 | 0.3865 | 0 | 0.91 | 0.3578 |
| GO:0009535 | chloroplast thylakoid membrane | 16 | 0 | 0.34 | 0.2169 | 3 | 2.88 | 0.354 | 0 | 2.42 | 0.4625 |
| GO:0016604 | nuclear body | 7 | 0 | 0.15 | 0.2197 | 3 | 1.26 | 0.3465 | 1 | 1.06 | 0.323 |
| GO:0044428 | nuclear part | 56 | 1 | 1.18 | 0.2222 | 12 | 10.07 | 0.2515 | 7 | 8.48 | 0.7348 |
| GO:0044430 | cytoskeletal part | 12 | 0 | 0.25 | 0.2282 | 0 | 2.16 | 0.4921 | 0 | 1.82 | 0.4471 |
| GO:0044434 | chloroplast part | 77 | 1 | 1.62 | 0.2358 | 9 | 13.85 | 0.5995 | 5 | 11.66 | 0.4765 |
| GO:0031967 | organelle envelope | 68 | 0 | 1.43 | 0.2493 | 9 | 12.23 | 0.3158 | 3 | 10.29 | 0.5932 |
| GO:0043226 | organelle | 643 | 11 | 13.56 | 0.2495 | 118 | 115.68 | 0.646 | 96 | 97.33 | 0.3621 |
| GO:0043229 | intracellular organelle | 643 | 11 | 13.56 | 0.2495 | 118 | 115.68 | 0.646 | 96 | 97.33 | 0.3621 |
| GO:0016021 | integral component of membrane | 60 | 2 | 1.27 | 0.2506 | 7 | 10.79 | 0.3199 | 6 | 9.08 | 0.3186 |
| GO:0009532 | plastid stroma | 41 | 1 | 0.86 | 0.2523 | 3 | 7.38 | 0.7099 | 4 | 6.21 | 0.4323 |
| GO:0009570 | chloroplast stroma | 41 | 1 | 0.86 | 0.2523 | 3 | 7.38 | 0.7099 | 4 | 6.21 | 0.4323 |
| GO:0043234 | protein complex | 78 | 2 | 1.65 | 0.2553 | 17 | 14.03 | 0.4436 | 15 | 11.81 | 0.4549 |
| GO:0000151 | ubiquitin ligase complex | 13 | 0 | 0.27 | 0.2716 | 3 | 2.34 | 0.4053 | 2 | 1.97 | 0.3811 |
| GO:0031975 | envelope | 70 | 0 | 1.48 | 0.2824 | 9 | 12.59 | 0.4338 | 3 | 10.6 | 6.50E-01 |
| GO:0034357 | photosynthetic membrane | 19 | 0 | 0.4 | 0.2827 | 6 | 3.42 | 0.1112 | 3 | 2.88 | 0.2074 |
| GO:0044436 | thylakoid part | 19 | 0 | 0.4 | 0.2827 | 6 | 3.42 | 0.1112 | 3 | 2.88 | 0.2074 |
| GO:0043227 | membrane-bounded organelle | 632 | 11 | 13.33 | 0.2884 | 116 | 113.7 | 0.4603 | 94 | 95.66 | 0.2612 |
| GO:0043231 | intracellular membrane-bounded organelle | 632 | 11 | 13.33 | 0.2884 | 116 | 113.7 | 0.4603 | 94 | 95.66 | 0.2612 |
| GO:0016020 | membrane | 342 | 7 | 7.21 | 0.2895 | 66 | 61.53 | 0.3467 | 49 | 51.77 | 0.6585 |
| GO:0044455 | mitochondrial membrane part | 13 | 0 | 0.27 | 0.2918 | 2 | 2.34 | 0.1902 | 3 | 1.97 | 0.1447 |
| GO:0042651 | thylakoid membrane | 17 | 0 | 0.36 | 0.3115 | 4 | 3.06 | 0.2478 | 1 | 2.57 | 0.3897 |
| GO:0055035 | plastid thylakoid membrane | 17 | 0 | 0.36 | 0.3115 | 4 | 3.06 | 0.2478 | 1 | 2.57 | 0.3897 |
| GO:0005743 | mitochondrial inner membrane | 17 | 0 | 0.36 | 0.3185 | 3 | 3.06 | 0.1765 | 3 | 2.57 | 0.2167 |
| GO:0044462 | external encapsulating structure part | 6 | 1 | 0.13 | 0.3233 | 0 | 1.08 | 0.5939 | 1 | 0.91 | 0.5024 |
| GO:0043233 | organelle lumen | 63 | 1 | 1.33 | 0.3239 | 15 | 11.33 | 0.1907 | 9 | 9.54 | 0.5808 |
| GO:0070013 | intracellular organelle lumen | 63 | 1 | 1.33 | 0.3239 | 15 | 11.33 | 0.1907 | 9 | 9.54 | 0.5808 |
| GO:0005777 | peroxisome | 29 | 1 | 0.61 | 0.33 | 5 | 5.22 | 0.5088 | 4 | 4.39 | 0.3447 |
| GO:0042579 | microbody | 29 | 1 | 0.61 | 0.33 | 5 | 5.22 | 0.5088 | 4 | 4.39 | 0.3447 |
| GO:0005740 | mitochondrial envelope | 19 | 0 | 0.4 | 0.3368 | 3 | 3.42 | 0.3376 | 3 | 2.88 | 0.2615 |
| GO:0046658 | anchored component of plasma membrane | 13 | 0 | 0.27 | 0.3576 | 3 | 2.34 | 0.5296 | 4 | 1.97 | 0.3348 |
| GO:0044429 | mitochondrial part | 22 | 0 | 0.46 | 0.3708 | 3 | 3.96 | 0.4372 | 3 | 3.33 | 0.3509 |
| GO:0005730 | nucleolus | 34 | 1 | 0.72 | 0.3826 | 7 | 6.12 | 0.2776 | 5 | 5.15 | 0.5097 |
| GO:0005783 | endoplasmic reticulum | 55 | 0 | 1.16 | 0.3891 | 6 | 9.89 | 0.5116 | 2 | 8.33 | 0.634 |
| GO:0044425 | membrane part | 119 | 3 | 2.51 | 0.3898 | 23 | 21.41 | 0.3073 | 22 | 18.01 | 0.3506 |
| GO:0005856 | cytoskeleton | 13 | 0 | 0.27 | 0.3907 | 0 | 2.34 | 0.714 | 0 | 1.97 | 0.6658 |
| GO:0005576 | extracellular region | 94 | 7 | 1.98 | 0.3961 | 27 | 16.91 | 0.0379 | 21 | 14.23 | 0.1788 |
| GO:0031974 | membrane-enclosed lumen | 65 | 1 | 1.37 | 0.4024 | 15 | 11.69 | 0.2788 | 9 | 9.84 | 0.6063 |
| GO:0031966 | mitochondrial membrane | 18 | 0 | 0.38 | 0.4111 | 3 | 3.24 | 0.2617 | 3 | 2.72 | 0.3149 |
| GO:0009504 | cell plate | 7 | 1 | 0.15 | 0.4134 | 2 | 1.26 | 0.6234 | 1 | 1.06 | 0.6824 |
| GO:0005623 | cell | 782 | 16 | 16.49 | 0.4172 | 142 | 140.68 | 0.4885 | 119 | 118.37 | 0.1563 |
| GO:0044464 | cell part | 782 | 16 | 16.49 | 0.4172 | 142 | 140.68 | 0.4885 | 119 | 118.37 | 0.1563 |
| GO:0031225 | anchored component of membrane | 17 | 0 | 0.36 | 0.4269 | 4 | 3.06 | 0.3528 | 5 | 2.57 | 0.136 |
| GO:0031461 | cullin-RING ubiquitin ligase complex | 7 | 0 | 0.15 | 0.4312 | 0 | 1.26 | 0.6172 | 0 | 1.06 | 0.5972 |
| GO:0043228 | non-membrane-bounded organelle | 82 | 1 | 1.73 | 0.4341 | 9 | 14.75 | 0.6574 | 8 | 12.41 | 0.6406 |
| GO:0043232 | intracellular non-membrane-bounded organelle | 82 | 1 | 1.73 | 0.4341 | 9 | 14.75 | 0.6574 | 8 | 12.41 | 0.6406 |
| GO:0031988 | membrane-bounded vesicle | 27 | 1 | 0.57 | 0.4447 | 6 | 4.86 | 0.5285 | 5 | 4.09 | 0.6011 |
| GO:0044431 | Golgi apparatus part | 7 | 0 | 0.15 | 0.4562 | 1 | 1.26 | 0.5772 | 0 | 1.06 | 0.5573 |
| GO:0009534 | chloroplast thylakoid | 21 | 0 | 0.44 | 0.4823 | 4 | 3.78 | 0.474 | 2 | 3.18 | 0.3755 |
| GO:0031976 | plastid thylakoid | 21 | 0 | 0.44 | 0.4823 | 4 | 3.78 | 0.474 | 2 | 3.18 | 0.3755 |
| GO:0031984 | organelle subcompartment | 21 | 0 | 0.44 | 0.4823 | 4 | 3.78 | 0.474 | 2 | 3.18 | 0.3755 |
| GO:0005773 | vacuole | 85 | 1 | 1.79 | 0.5346 | 15 | 15.29 | 0.748 | 6 | 12.87 | 0.9042 |
| GO:0031982 | vesicle | 28 | 1 | 0.59 | 0.5388 | 6 | 5.04 | 0.567 | 5 | 4.24 | 0.7094 |
| GO:0005739 | mitochondrion | 150 | 1 | 3.16 | 0.5423 | 25 | 26.99 | 0.599 | 18 | 22.7 | 0.8892 |
| GO:0044445 | cytosolic part | 19 | 0 | 0.4 | 0.5646 | 1 | 3.42 | 0.9784 | 0 | 2.88 | 0.9971 |
| GO:0005774 | vacuolar membrane | 47 | 0 | 0.99 | 0.5751 | 6 | 8.46 | 0.7122 | 2 | 7.11 | 0.7122 |
| GO:0005886 | plasma membrane | 208 | 7 | 4.39 | 0.5768 | 48 | 37.42 | 0.0591 | 33 | 31.48 | 0.5611 |
| GO:0012505 | endomembrane system | 25 | 0 | 0.53 | 0.5777 | 1 | 4.5 | 0.8065 | 1 | 3.78 | 0.5385 |
| GO:0044437 | vacuolar part | 50 | 0 | 1.05 | 0.5867 | 8 | 9 | 0.67 | 3 | 7.57 | 0.6959 |
| GO:0031226 | intrinsic component of plasma membrane | 14 | 0 | 0.3 | 0.6117 | 4 | 2.52 | 0.4796 | 5 | 2.12 | 0.2092 |
| GO:0015934 | large ribosomal subunit | 9 | 0 | 0.19 | 0.6142 | 0 | 1.62 | 0.8325 | 0 | 1.36 | 0.7974 |
| GO:0030529 | ribonucleoprotein complex | 38 | 0 | 0.8 | 0.6178 | 3 | 6.84 | 0.9337 | 3 | 5.75 | 0.7078 |
| GO:0005770 | late endosome | 6 | 0 | 0.13 | 0.621 | 1 | 1.08 | 0.7159 | 0 | 0.91 | 0.7016 |
| GO:0044444 | cytoplasmic part | 527 | 10 | 11.12 | 0.6465 | 93 | 94.81 | 0.8717 | 78 | 79.77 | 0.8363 |
| GO:0005794 | Golgi apparatus | 64 | 1 | 1.35 | 0.6623 | 13 | 11.51 | 0.4251 | 7 | 9.69 | 0.9451 |
| GO:0005840 | ribosome | 33 | 0 | 0.7 | 0.6687 | 2 | 5.94 | 0.9541 | 2 | 5 | 0.8968 |
| GO:0005654 | nucleoplasm | 17 | 0 | 0.36 | 0.6714 | 4 | 3.06 | 0.9785 | 2 | 2.57 | 0.9767 |
| GO:0071944 | cell periphery | 239 | 7 | 5.04 | 0.6835 | 60 | 43 | 0.127 | 42 | 36.18 | 0.2783 |
| GO:0044459 | plasma membrane part | 26 | 1 | 0.55 | 0.6975 | 7 | 4.68 | 0.4666 | 8 | 3.94 | 0.1594 |
| GO:0000325 | plant-type vacuole | 9 | 0 | 0.19 | 0.7051 | 0 | 1.62 | 0.801 | 0 | 1.36 | 0.8089 |
| GO:0032991 | macromolecular complex | 117 | 2 | 2.47 | 0.7073 | 21 | 21.05 | 0.8815 | 19 | 17.71 | 0.8242 |
| GO:0005694 | chromosome | 10 | 0 | 0.21 | 0.7147 | 1 | 1.8 | 0.8531 | 2 | 1.51 | 0.6087 |
| GO:0005768 | endosome | 27 | 0 | 0.57 | 0.7165 | 5 | 4.86 | 0.4789 | 3 | 4.09 | 0.7938 |
| GO:0022626 | cytosolic ribosome | 18 | 0 | 0.38 | 0.7311 | 1 | 3.24 | 0.9972 | 0 | 2.72 | 0.9972 |
| GO:0005737 | cytoplasm | 577 | 12 | 12.17 | 0.7491 | 100 | 103.8 | 0.8877 | 87 | 87.34 | 0.6565 |
| GO:0031410 | cytoplasmic vesicle | 25 | 0 | 0.53 | 0.751 | 4 | 4.5 | 0.8391 | 5 | 3.78 | 0.7004 |
| GO:0044391 | ribosomal subunit | 11 | 0 | 0.23 | 0.7517 | 0 | 1.98 | 0.9461 | 0 | 1.67 | 0.9203 |
| GO:0005802 | trans-Golgi network | 18 | 0 | 0.38 | 0.7575 | 5 | 3.24 | 0.2689 | 2 | 2.72 | 0.591 |
| GO:0016023 | cytoplasmic membrane-bounded vesicle | 24 | 0 | 0.51 | 0.7602 | 4 | 4.32 | 0.8452 | 5 | 3.63 | 0.6714 |
| GO:0015630 | microtubule cytoskeleton | 7 | 0 | 0.15 | 0.7859 | 0 | 1.26 | 0.9569 | 0 | 1.06 | 0.9569 |
| GO:0048046 | apoplast | 42 | 1 | 0.89 | 0.7985 | 9 | 7.56 | 0.6943 | 7 | 6.36 | 0.8434 |
| GO:0022625 | cytosolic large ribosomal subunit | 7 | 0 | 0.15 | 0.8622 | 0 | 1.26 | 0.981 | 0 | 1.06 | 0.9679 |
| GO:0009295 | nucleoid | 6 | 0 | 0.13 | 0.876 | 0 | 1.08 | 0.9472 | 1 | 0.91 | 0.7649 |
| GO:0009507 | chloroplast | 198 | 3 | 4.18 | 0.934 | 30 | 35.62 | 0.9093 | 25 | 29.97 | 0.9935 |
| GO:0009536 | plastid | 238 | 4 | 5.02 | 0.967 | 38 | 42.82 | 0.905 | 34 | 36.02 | 0.9172 |
| GO:0044451 | nucleoplasm part | 14 | 0 | 0.3 | 0.9695 | 3 | 2.52 | 0.9843 | 1 | 2.12 | 0.9831 |
| GO:0005575 | cellular_component | 806 | 17 | 17 | 1 | 145 | 145 | 1 | 122 | 122 | 1 |

Note:GO ID indicate the ID of GO term；Term indicate GO gunction；Annotated indicate the annotated genes numbers in all genes；Significant indicate the annotated DEGs number；Expected indicate the expect value for the annotated DEGs；KS indicate the significant statistics of enriched terms, the smaller KS value shows higher significanlt enrichment.
