# Supplementary material for: Identification of Key Gene Networks and Deciphering Transcriptional Regulators Associated With Peanut Embryo Abortion Mediated by Calcium Deficiency
Source: Front Plant Sci. 2022 Mar 21;13:814015. doi: 10.3389/fpls.2022.814015 (PMC8978587; doi:10.3389/fpls.2022.814015)
Supplement: Supplementary file 10 [file Table_6.docx]

**Supplementary Table 6 KEGG pathway enrichment of DEGs**

| **Stages** | **# Pathway** | **KO** | **Enrichment_Factor** | **Q-value** |
| --- | --- | --- | --- | --- |
| 15 DAP | Photosynthesis - antenna proteins | ko00196 | 33.34 | 0.00 |
|  | Glycolysis / Gluconeogenesis | ko00010 | 3.34 | 0.00 |
|  | Photosynthesis | ko00195 | 3.90 | 0.00 |
| 20 DAP | Photosynthesis - antenna proteins | ko00196 | 4.47 | 0.00 |
|  | Flavonoid biosynthesis | ko00941 | 3.50 | 0.00 |
|  | Fatty acid biosynthesis | ko00061 | 2.64 | 0.00 |
|  | Glycolysis / Gluconeogenesis | ko00010 | 1.71 | 0.00 |
|  | Porphyrin and chlorophyll metabolism | ko00860 | 2.12 | 0.00 |
|  | Starch and sucrose metabolism | ko00500 | 1.48 | 0.00 |
|  | Phenylpropanoid biosynthesis | ko00940 | 1.60 | 0.01 |
|  | Amino sugar and nucleotide sugar metabolism | ko00520 | 1.49 | 0.01 |
|  | Plant-pathogen interaction | ko04626 | 1.47 | 0.03 |
|  | Diterpenoid biosynthesis | ko00904 | 2.63 | 0.03 |
|  | Phenylalanine metabolism | ko00360 | 1.62 | 0.04 |
|  | Vitamin B6 metabolism | ko00750 | 3.10 | 0.05 |
| 30 DAP | Photosynthesis - antenna proteins | ko00196 | 5.40 | 0.00 |
|  | Glycolysis / Gluconeogenesis | ko00010 | 1.98 | 0.00 |
|  | Fatty acid biosynthesis | ko00061 | 2.94 | 0.00 |
|  | Porphyrin and chlorophyll metabolism | ko00860 | 2.13 | 0.00 |
|  | Carbon fixation in photosynthetic organisms | ko00710 | 1.79 | 0.00 |
|  | Starch and sucrose metabolism | ko00500 | 1.52 | 0.00 |
|  | Pyruvate metabolism | ko00620 | 1.64 | 0.00 |
|  | Galactose metabolism | ko00052 | 2.07 | 0.00 |
|  | Nitrogen metabolism | ko00910 | 2.10 | 0.00 |
|  | Terpenoid backbone biosynthesis | ko00900 | 1.91 | 0.01 |
|  | Taurine and hypotaurine metabolism | ko00430 | 3.21 | 0.01 |
|  | Alanine, aspartate and glutamate metabolism | ko00250 | 1.88 | 0.02 |
|  | Flavonoid biosynthesis | ko00941 | 2.40 | 0.04 |
