# Supplementary material for: Identification of Key Gene Networks and Deciphering Transcriptional Regulators Associated With Peanut Embryo Abortion Mediated by Calcium Deficiency
Source: Front Plant Sci. 2022 Mar 21;13:814015. doi: 10.3389/fpls.2022.814015 (PMC8978587; doi:10.3389/fpls.2022.814015)
Supplement: Supplementary file 15 [file Table_11.docx]

**Supplementary Table 11 Specific primer sequences used for qRT-PCR**

| **Gene Name** | **Forward primer** | **Reverse primer** |
| --- | --- | --- |
| *APIFA* | ACTGGCCACTCAGAACCTC | AGCCACTTCAGTGTCTTCC |
| *EMB2781* | AGCATGTATTGTCTAGAGAGC | ACTCAAACCATGAGAAACCTC |
| *XTH23* | TGTTGCTGGCAACTCTGCTG | ACTGTTGCTCTCTGTCACC |
| *CYP707A3* | AGGATGTTAGGGAAGCAAGC | TGATCAAACGTCCTTGGCAAG |
| *CYP707A4* | AGCTCATAGGTCCTTCTGC | ACACTGAGAGGATTCCAACG |
| *CPK28* | TGCTCAGGCTTTGTCACATC | TGTCTCATCTCTTCCAGAC |
| *ANT* | AGGCATAGGTGGACTGGTAG | AGAAGGTCATTGCTTGCCA |
| *WRI1* | TGGAAACATATGCCAAGG | ACTCTTCCAATTCGTGC |
| *ABI5-1* | TCATTCTGTGTCATCCCATC | TGTTGCTTTCTAAGTGCCTC |
| *TCP4* | TCCAGTTCTACGACGTTCAG | AGAAGCTTCCATAGCGTCG |
| *FUS3* | TCACCAACACTGATGCACCTTC | TCCTGAGAGAACTAACATCACTG |
| *MYB113* | TTGGCACTTCAAGCTCCATG | TCCATAGGCAACTCACCAAC |
| *Ahactin* | GAGGAGAATCAGAAGCAAGTC | CATATACAGCATAGCGGCACTC |
